# *Hypericum empetrifolium* subsp. *empetrifolium*: an assessment of its antifungal, antidiabetic, anti-aging, and neuroprotective potential

**DOI:** 10.3389/fphar.2025.1618761

**Published:** 2025-07-07

**Authors:** Serpil Demirci Kayıran, Ömerül Faruk Tavlı, Emel Mataracı Kara, Alevcan Kaplan, Hasan Şahin, Mehmet Boğa, Esra Eroğlu Özkan

**Affiliations:** ^1^ Department of Pharmaceutical Botany, Faculty of Pharmacy, Çukurova University, Adana, Türkiye; ^2^ Department of Pharmacognosy, Faculty of Pharmacy, Biruni University, Istanbul, Türkiye; ^3^ Department of Pharmaceutical Microbiology, Faculty of Pharmacy, Istanbul University, Istanbul, Türkiye; ^4^ Department of Crop and Animal Production, Sason Vocational School, Batman University, Batman, Türkiye; ^5^ Department of Pharmacognosy, Faculty of Pharmacy, Dicle University, Diyarbakır, Türkiye; ^6^ Department of Analytical Chemistry, Faculty of Pharmacy, Dicle University, Diyarbakır, Türkiye; ^7^ Department of Pharmacognosy, Faculty of Pharmacy, Istanbul University, Istanbul, Türkiye

**Keywords:** *Hypericum empetrifolium* subsp. *empetrifolium* Willd, anti-candida, anti-cholinesterase, anti-α-glucosidase, anti-aging, HPLC, LC-HR/MS

## Abstract

**Introduction:**

*Hypericum empetrifolium* subsp. *empetrifolium* Willd. (Hypericaceae), traditionally used in folk medicine, was investigated for its diverse biological activities. The study was driven by the increasing global health and economic burden posed by fungal infections, highlighting the urgent need for novel antifungal agents.

**Methods:**

Plant materials were collected from distinct regions in Türkiye. Methanolic extracts were prepared and tested in vitro for antimicrobial and enzyme inhibition activities. Minimum inhibitory concentration (MIC) and IC_50_ values were determined using standard microdilution and spectrophotometric assays. Phytochemical profiling of the extracts was performed using HPLC-DAD and LC-HR/MS to identify major chemical constituents and investigate variation due to geographic and environmental factors.

**Results:**

Methanolic extracts exhibited potent antifungal activity against *Candida* strains, with MIC values as low as 4.88 μg/mL. Enzyme inhibition assays revealed strong activity for HE-1, with IC_50_ values of 8.16 μg/mL for acetylcholinesterase and 2.46 μg/mL for butyrylcholinesterase. The extracts also significantly inhibited α-glucosidase (IC_50_ ≈ 26–31 μg/mL), outperforming acarbose, and showed moderate inhibition against elastase. Phytochemical profiling indicated notable variations in flavonoid and phenolic acid content, likely influenced by geographic and environmental factors.

**Discussion:**

These findings suggest that *H. empetrifolium* subsp. *empetrifolium* extracts possess promising antifungal, antidiabetic, anti-aging, and neuroprotective properties. The observed bioactivities and phytochemical richness support further exploration of this species as a potential source of therapeutic agents.

## 1 Introduction

In 2019 alone, the economic burden of fungal disease-related illnesses and deaths in the United States was estimated to exceed USD 24.3 billion (Kumar et al., 2022; Benedict et al., 2022). Invasive candidiasis and aspergillosis accounted for 30 % of this cost, while noninvasive candidiasis, a widespread infection, also contributed a significantly (Benedict et al., 2022).

Invasive candidiasis is a severe fungal infection that can affect various organs, often occurring in immunocompromised patients with weakened host defense mechanisms (Lass-Flörl et al., 2024). The presence of invasive candidiasis is associated with a high mortality rates, with an attributable death rate of 49 % which can rise to 98 % in patients with septic shock and delayed antifungal treatment (Thomas-Rüddel et al., 2022). The most critical form of invasive candidiasis, candidemia, represents a major cause hospital-acquired bloodstream infections, surpassing some bacterial pathogens, such as *Pseudomonas aeruginosa* (Timsit et al., 2020). *Candida* species are among the most common and serious infectious agents in intensive care units (IUCs), with candidemia cases rank fifth in IUCs in the United States and Europe, highlighting the substantial threat posed by *Candida* infections (Selvan et al., 2022). While *Candida albicans* remains the most prevalent causative species, infections caused by multidrug-resistant non-*Candida albicans* species, including *C. parapsilosis, C. tropicalis, C. glabrata* (*Nakaseomyces glabratus*), *C. krusei* and *C. auris*, are increasingly concerning (Mamali et al., 2022; Pfaller et al., 2019; Pappas et al., 2018). The incidence of non-*Candida albicans* species has been rising globally (Pappas et al., 2018; Vallabhaneni et al., 2015; Lortholary et al., 2011). Furthermore, according to the 2019 Antibiotic Resistance Threats in the United States report by the CDC (Centers for Disease Control and Prevention), drug-resistant *Candida* species were responsible for 34,800 infections and 1,700 deaths (Pianalto and Alspaugh, 2016; Mota Fernandes et al., 2021).

Beyond drug resistance, existing antifungal agents—including polyenes (e.g., amphotericin B), triazoles (e.g., fluconazole), and echinocandins (e.g., caspofungin)—exhibit high toxicity and a narrow therapeutic index. Their limited oral bioavailability significantly restricts treatment efficacy (Hasim and Coleman, 2019; Gómez-López, 2020). Broad-spectrum triazoles, such as posaconazole and voriconazole, face further limitations due to drug-drug interactions, variable bioavailability, acute adverse events, and emerging resistance (Miceli and Kauffman, 2015).

In addition to systemic antifungals, topical imidazole derivatives such as clotrimazole remain a mainstay for superficial candidiasis. Clotrimazole exerts fungistatic and fungicidal effects via inhibition of ergosterol biosynthesis, yet it presents notable limitations including poor systemic bioavailability, limited tissue penetration, and the need for frequent application, which can compromise patient adherence (Ivanov et al., 2022; Zhou et al., 2016). Although resistance is relatively rare, emerging cases—particularly involving non-*Candida albicans* species—have been reported, often associated with efflux pump overexpression and ERG11 mutations (Mendling et al., 2020; Keshwania et al., 2023). Local adverse effects such as burning and irritation may also reduce tolerability (Hajizadeh et al., 2025). While clotrimazole remains a valuable agent for localized infections, its pharmacokinetic constraints and formulation-dependent efficacy necessitate cautious use in complicated or recurrent cases (Mendling et al., 2020; Kaur and Kakkar, 2010). These challenges highlight the urgent need for novel antifungal agents with improved efficacy and safety profiles.

*Hypericum empetrifolium* subsp. *empetrifolium* Willd. (Hypericaceae) belongs to the section Coridium, which comprises six species (Robson, 1981; Royal Botanic Gardens, Kew, 2025). This shrub thrives in rocky terrains and is widely distributed at low altitudes throughout the Aegean region, particularly in southern mainland Greece and the coastal areas of western Türkiye (Trovato et al., 2001). In Türkiye, *H. empetrifolium* subsp. *empetrifolium* is locally known as “sarı piren” or “piren” (“sarı” meaning yellow), and its flower decoctions have been traditionally used for dyeing cloth in western Anatolia (Baytop, 1999). Additionally, the species has been reported to possess significant medicinal properties, including applications for kidney stones and gastric ulcers (Crockett et al., 2008), as well as for treating burn wounds. It also exhibits antispasmodic, laxative, anthelmintic, and antiseptic effects (Ozturk et al., 2013).

Our previous research, as part of an ongoing project investigating plant-derived antimicrobial natural products from the genus *Hypericum*, demonstrated that the ethanolic extract of the aerial parts of *H. empetrifolium* exhibited antifungal activity against *C. parapsilosis* (MIC = 4.88 μ g/mL), *C. tropicalis* (MIC = 19.53 μ g/mL), and *C. albicans* (MIC = 78.12 μ g/mL). Phytochemical analysis revealed that the ethanolic extract was rich in chlorogenic acid, isoquercitrin, malic acid, protocatechuic acid, quercetin, and fumaric acid. Additionally, several phenolic metabolites, including salicylic acid, caffeic acid, p-coumaric acid, rutin, nicotiflorin, rosmarinic acid, naringenin, apigenin, and vanillin, were identified in lower concentrations (Bo et al., 2021).

Apart from our previous findings, other research groups have also reported various bioactivities of *H. empetrifolium*. Couladis et al. reported the methanolic extract of the plant having cytotoxic effect in human colon carcinoma and human hepatoma cell lines (Couladis et al., 2002). Trovato and colleagues demonstrated the methanolic extract of the plant’s aerial parts exhibited a significant anti-inflammatory activity and analgesic effects *in vivo* writhing test (Trovato et al., 2001). Subsequently, Crockett et al. isolated two acylphloroglucinols with moderate to potent *in vitro* activity against COX-1, COX-2, and 5-LOX (Crockett et al., 2008). Schmidt et al. isolated phloroglucinol derivatives from the petroleum ether extract of the plant, which exhibited *in vitro* antiproliferative activity against human microvascular endothelial cells (HMEC-1) (Schmidt et al., 2012c; b). Additionally, phytochemical investigations of methanolic extract of Jordanian *H. empetrifolium* identified hypericin and hyperforin (Tawaha et al., 2010) as well as pseudohypericin, protohypericin, and adhyperfirin (Alali et al., 2009).

In this study, three samples of *H. empetrifolium* subsp. *empetrifolium* (*sect.* Coridium) were collected from different regions in Türkiye and their methanolic extracts were analyzed using HPLC-DAD and LC-HR/MS, respectively. Given that certain key metabolites, such as naphthodianthrones and phloroglucinols, are highly lipophilic and exhibit greater solubility in organic alcohols, methanol was selected as the extraction solvent, as previously recommended (Zeliou et al., 2020). The total phenolic and flavonoid content, antioxidant capacity (i.e., ABTS, DPPH, and CUPRAC). Additionally, antimicrobial activity was evaluated using the microdilution method to determine the antimicrobial potential of *H. empetrifolium* subsp. *empetrifolium* against standard yeast, Gram (−), and Gram (+) bacterial strains, and enzyme inhibitory activities, including anti-acetylcholinesterase, anti-butyrylcholinesterase, anti- α -Glucosidase, anti-tyrosinase, and anti-aging (anti-hyaluronidase and anti-elastase) activities, were assessed using ELISA reader-based methods. This study aimed to compare the metabolic profiles of *H. empetrifolium* subsp. *empetrifolium* growing in the wild habitats and conditions, along with their antimicrobial and other biological activities.

This study was conducted to address the urgent need for safer and more effective antifungal agents by investigating the phytochemical richness and bioactivity of *H. empetrifolium* subsp. *empetrifolium* collected from different ecological regions of Türkiye. As highlighted above, current antifungal treatments are constrained by toxicity, resistance, and poor bioavailability. In contrast, the demonstrated antifungal and enzyme inhibitory properties of the extracts underscore the therapeutic potential of this species. Notably, *H. empetrifolium* subsp. *empetrifolium* has received limited attention in the current literature. Therefore, this research not only expands the phytochemical and pharmacological knowledge of this underexplored species but also fills a critical gap in the literature by linking its diverse bioactivities with ecological variation.

## 2 Materials and methods

### 2.1 Preparation of plant extracts

Aerial parts of *H. empetrifolium* subsp. *empetrifolium* were collected during the flowering stage from Sandras Mountain, in the Denizli region (HE-1, CUEF 1761; HE-2, CUEF 1762) and Geyik Mountains, in the Konya region (HE-3, CUEF1763) in June 2022, and determined by Dr. Serpil Demirci Kayıran who is an associate professor at Çukurova University Faculty of Pharmacy Department of Pharmaceutical Botany. Voucher specimens were deposited in the Herbarium of Çukurova University Faculty of Pharmacy (Türkiye).

Methanol was chosen as the extraction solvent due to its well-documented efficiency in recovering both polar and moderately lipophilic secondary metabolites, including phenolic acids, flavonoids, naphtodianthrones, and phloroglucinol derivatives, which are known to be abundant in *Hypericum* species (Ion et al., 2022).

The methanolic extract of *H. empetrifolium* subsp. *empetrifolium* aerial parts were used for the phytochemical analysis and biological assays. 10 g of crushed aerial parts of *H. empetrifolium* subsp. *empetrifolium* was macerated with 100 mL of analytical-grade methanol. The extracts were filtered and the solvent removed *in vacuo*, and the residue was lyophilized. The extraction yields were calculated as 13.15 % (g_crude extract_/g_crushed plant_) for HE-1, 21.18 % (g_crude extract_/g_crushed plant_) for HE-2, and 16.68 % (g_crude extract_/g_crushed plant_) for HE-3, respectively.

### 2.2 Chemical analysis

#### 2.2.1 HPLC-DAD analysis

Quantitative analysis was conducted following the European Pharmacopoeia method for the evaluation of hypericin, pseudohypericin, and hyperforin (European Pharmacopoeia, 2008). The analysis was performed on a methanolic extract using an HPLC-DAD system (Shimadzu model 20A, Shimadzu Analytical and Measuring Instruments, Kyoto, Japan), equipped with a pump (LC-20AD), a diode array detector (DAD) (SPD-M20A), and an autosampler (SIL-20AD). Chromatographic separation was achieved using a Thermo-Fisher C18 column (250 × 4.6 mm i.d., 5 μ m particle size, USA).

The operational conditions for pseudohypericin and hypericin were as follows: a flow rate of 1 mL/min, a column oven temperature of 40°C, an injection volume of 20 μ L, and a detection wavelength of 590 nm. For hyperforin, the conditions included a flow rate of 1 mL/min, a column oven temperature of 40°C, an injection volume of 10 μ L, and a detection wavelength of 275 nm.

For the identification and quantification of pseudohypericin and hypericin, an isocratic solvent system was employed, consisting of solvent A [ethyl acetate/15.6 g/L sodium dihydrogen phosphate (adjusted to pH 2 with phosphoric acid)/methanol (39:41:160, v/v/v)]. The gradient solvent system used for phenolic metabolites and hyperforin consisted of Mobile Phase A (0.3 % formic acid in water, v/v) and Mobile Phase B (0.3 % formic acid in acetonitrile, v/v), with the following gradient profile: 18 % B (0–8 min), 18 →53% B (8–18 min), 53 →97% B (18–18.1 min), 97 % B (18.1–19 min), 97 % B (19–29 min), 97 →18% B (29–30 min). All solvents were filtered through a 0.45 μ m filter and degassed in an ultrasonic bath before use. System control and data analysis were conducted using Shimadzu LC Solutions software.

#### 2.2.2 LC-HR/MS analysis

A previously validated method (Kino et al., 2023) was employed with minor revisions to identify the phenolic constituents of the extracts. For sample preparation, 100 mg of dried extract was diluted with 1.8 mL of methanol, followed by the addition of 0.2 mL of an internal standard solution to obtain a final concentration of 50 mg/mL. The samples were then filtered through a 0.45 μ m membrane filter before analysis.

LC-HR/MS analysis was performed using a Thermo ORBITRAP Q-EXACTIVE system (Bremen, Germany). Chromatographic separation was achieved on a Fortis UniverSil C18 analytical column (150 mm × 3 mm, 3 μ m, United Kingdom), with the column oven temperature set at 40°C. The mobile phases consisted of Mobile Phase A (1 % formic acid in ultrapure water, v/v) and Mobile Phase B (1 % formic acid in methanol, v/v). The following LC gradient elution program was applied for optimal separation: 50 % B (0–1 min), 50 →100% B (1–3 min), 100 % B (3–6 min), 100 →50% B (6–7 min), 50 % B (7–10 min). The system operated at a constant flow rate of 0.35 mL/min, with an injection volume of 2 μ L.

Metabolites identification was performed by comparing the retention times of reference standards (95–99 % purity) with high-resolution mass spectrometry (HR/MS) data from the Bezm-i Alem Vakıf University Drug Application and Research Center Library (ILMER). To enhance measurement repeatability and mitigate variations caused by external factors such as ionization fluctuations in mass spectrometry, dihydrocapsaicin (95 % purity) was used as an internal standard in LC-HR/MS analysis.

### 2.3 Chemical tests assessing radical scavenging capacity

#### 2.3.1 Determination of total phenolic content

The total phenolic content of the extracts was quantified using the Folin-Ciocalteu reagent, with pyrocatechol as the standard. A standard solution of pyrocatechol was prepared at 100 ppm concentration. Aliquots of this solution, in volumes of 0–8 μ L, were diluted to a total volume of 184 μ L with distilled water (Milli-Q). Similarly, the extracts were prepared at a concentration of 1 mg/mL, from which 4 μ L samples were taken and brought up to 184 μ L with distilled water. To each of these dilutions, 4 μ L of Folin-Ciocalteu reagent was added, followed by 12 μ L of a 2 % Na_2_CO_3_ solution after a 3-min interval. The resulting mixtures were incubated for 2 h at room temperature in the dark. Absorbance was subsequently measured at 760 nm (Slinkard and Singleton, 1977).

#### 2.3.2 Determination of total flavonoid content

The total flavonoid content of the extracts was determined by the aluminum nitrate method, using quercetin as a standard. A quercetin solution was prepared at a concentration of 1,000 ppm. Serial dilutions of this solution, ranging from 0 to 8 μ L, were completed to 192 μ L with 80 % ethanol. To these dilutions, 4 μ L of 1 M potassium acetate and 4 μ L of a 10 % aluminum nitrate solution were added sequentially. The mixtures were incubated for 40 min at room temperature. Absorbance was then read at 415 nm using BioTek Power Wave XS microplate photometer. The same procedure was applied to the extracts prepared at a concentration of 1,000 ppm to determine their flavonoid content (Moreno et al., 2000).

#### 2.3.3 1,1-Diphenyl-2-picrylhydrazyl (DPPH) free radical scavenging assay

The DPPH radical scavenging assay was evaluated using 1,1-diphenyl-2-picrylhydrazyl (DPPH) free radical according to the method developed by Blois (1958). Extracts were dissolved in methanol at a concentration of 1 mg/mL to prepare the primary stock solutions. Aliquots of these solutions, in volumes of 2, 5, 10, and 20 μ L, were each diluted to a final volume of 40 μ L with methanol. Subsequently, 160 μ L of DPPH solution at a 0.1 mM concentration was introduced to each mixture. After the solutions were incubated at room temperature in the dark for 30 min, their absorbance was measured at a wavelength of 517 nm. BHA, BHT, and α -TOC were used as positive control.

#### 2.3.4 2,2′-azino-bis (3-ethylbenzothiazoline-6-sulfonic acid) (ABTS) cation scavenging assay

The ABTS cation radical scavenging assay was evaluated using 2,2′-azino-bis(3-ethylbenzothiazoline-6-sulfonic acid) according to the method developed by Re et al. (1999). Extracts were dissolved in methanol at a concentration of 1 mg/mL to prepare the primary stock solutions. Aliquots of these solutions, in volumes of 2, 5, 10, and 20 μ L, were each diluted to a final volume of 40 μ L with methanol. Subsequently, 160 μ L of ABTS cation radical solution at a 7 mM concentration was introduced to each mixture. The reactions were allowed to proceed in darkness for 6 min, subsequent to which the absorbance was recorded at a wavelength of 734 nm. BHA, BHT, and α -TOC were used as positive control.

#### 2.3.5 Cupric reducing antioxidant capacity (CUPRAC) method

In the CUPRAC assay, the presence of antioxidative metabolites in the samples leads to the reduction of the Cu (II)-Neocuproine (Nc) complex to a Cu(I)-Nc chelate, exhibiting an orange-yellow hue. The absorbance of this chelate formation is then quantified at a wavelength of 450 nm. In the CUPRAC method, according to the procedure developed by Apak et al.; Cu (II), neocuproine (2,9-dimethyl-1,10-phenanthroline), and NH_4_OAc buffer were added to the sample and standard solutions to achieve final concentrations of 10, 25, 50, and 100 μ g/mL. After an incubation period of 1 h, the absorbance was measured at 450 nm. BHA, BHT, and α -TOC were used as positive control (Apak et al., 2004).

### 2.4 Antimicrobial activity asssays

The Minimum Inhibitory Concentrations (MIC) of the extracts were determined using the broth microdilution method in accordance with the guidelines of the Clinical and Laboratory Standards Institute (CLSI) (Clinical and Laboratory Standards Institute, 2020). The procedure was originally based on the CLSI M27-A2 standard (1997) (Clinical and Laboratory Standards Institute, 2017) for antifungal susceptibility testing, while the updated fourth edition (2017) was referenced to reflect current methodological standards (Clinical and Laboratory Standards Institute, 1997).

The inocula of the tested bacterial and yeast strains were prepared following CLSI protocols, and the extracts were dissolved in DMSO to prepare stock solutions. Serial dilutions ranging from 1,250 to 0.06 μ g/mL were performed in Mueller-Hinton Broth (for bacterial strains) and RPMI-1640 medium (for yeast strains). MIC values of the extracts were determined against four g (−) bacterial strains (*P. aeruginosa* ATCC 27853, *Escherichia coli* ATCC 25922, *Klebsiella pneumoniae* ATCC 4352, *Proteus mirabilis* ATCC 14153), three g (+) bacterial strains (*Staphylococcus aureus* ATCC 29213, *Staphylococcus epidermidis* ATCC 12228, *Enterococcus faecalis* ATCC 29212), and three yeast strains (*Candida albicans* ATCC 10231, *Candida parapsilosis* ATCC 22019, *Candida tropicalis* ATCC 750).

### 2.5 Enzyme inhibition activity assays

#### 2.5.1 Anti-cholinesterase activity assays

The acetylcholinesterase and the butyrylcholinesterase inhibitory assays were conducted using a slightly modified method described by Ellman (Ozkok et al., 2022). Acetylthiocholine iodide (or butyrylthiocholine iodide) was used as substrate of the reaction and DTNB (5,5′ dithiobis nitrobenzoic acid) was used for the measurement of the anticholinesterase activity. 130 μ L of sodium phosphate buffer (pH 8.0), 10 μ L of 4 mM sample solution and 20 μ L of AChE (or BChE) solution were mixed in each well and incubated for 15 min at 25°C. The reaction commenced with the addition of 10 μ L of DTNB and 10 μ L of either acetylthiocholine iodide or butyrylthiocholine iodide. The tested solutions were at a final concentration of 200 μ g/mL. The hydrolysis of these substrates was monitored using BioTek Power Wave XS microplate photometer by the formation of yellow 5-thio-2-nitrobenzoate anion as the result of the reaction of DTNB with thiocholine, released by the enzymatic hydrolysis of acetylthiocholine iodide (or butyrylthiocholine iodide), at a wavelength of 412 nm. Galantamine was used as positive control.

#### 2.5.2 Anti-tyrosinase activity assay

Tyrosinase inhibitory assay was performed according to the method described by Hearing and Jimenez (1987). Initially, the ability of the metabolites to inhibit the diphenolase activity was assessed using L-DOPA as the substrate. Tyrosinase from mushroom (E.C. 1.14.18.1) (30 U, 28 nM) was dissolved in Na-phosphate buffer (pH = 6.8, 50 nM) and the compounds were added to the solution for pre-incubation at room temperature for 10 minutes. The enzymatic reaction was initiated by introducing 0.5 mM of L-DOPA into the mixture, followed by monitoring the absorbance shift at a wavelength of 475 nm at a temperature of 37°C. Kojic acid was used as positive control.

#### 2.5.3 Anti- α -glucosidase activity assay

A previously described method was used with minor changes for α -Glucosidase inhibitory assay (Schmidt J. et al., 2012). In brief, 10 μ L of the extracts dissolved in DMSO were added to the wells along with 90 μ L of phosphate buffer (pH 7.5) which was prepared using Na_2_HPO_4_, NaH_2_PO_4_, ultra-pure water (Milli-Q), and NaN_3_ (0.02 % ). 80 μ L of enzyme ( α -Glucosidase Type I, 0.05 U/mL) solution were added to each well. The mixture was incubated at 28°C for 10 min. Then, 20 μ L of the substrate (p-nitrophenol, α -D-glucopyranoside, 1.0 mM) was added to each well. The blank wells were consisted of the same mixture with buffer (DMSO 10 % ) instead of sample solutions. BioTek Power Wave XS microplate photometer was used for incubations and absorbance measurements at 405 nm. Photometer was set to read the absorbances in every 40 s for 35 min to obtain an absorbance/time graph. Slopes of the graphs were used to eliminate the potential in-fluence of the colored samples on absorbance. Acarbose was used as positive control.

#### 2.5.4 Antiaging activity assays

##### 2.5.4.1 Anti-hyaluronidase activity assay

The hyaluronidase inhibition assay was conducted using a sensitive spectrophotometric method developed by Tung et al. (1994). Hyaluronidase from bovine testes (E.C. 3.2.1.35) was dissolved in 50 mM Tris-HCl buffer (pH 7.0). Hyaluronic acid sodium salt was used as the substrate and prepared in the same buffer at a concentration of 0.4 mg/mL. The plant extracts were incubated with the enzyme solution at 37°C for 1 h. Following incubation, 10 % cetylpyridinium chloride solution was added. The final reaction mixture had a total volume of 110 μ L, consisting of 70 μ L Tris-HCl buffer, 10 μ L enzyme solution, 10 μ L substrate, 10 μ L cetylpyridinium chloride solution, and 10 μ L of the test extract. For the positive control, tannic acid (1.1 mg/mL) was used in place of the substrate. After incubation, absorbance was measured at 415 nm.

##### 2.5.4.2 Anti-elastase activity assays

The elastase inhibition assay was conducted using a spectrophotometric method developed by Lee et al. (1999). Porcine pancreatic elastase (E.C. 3.4.21.36) at a concentration of 3.33 mg/mL was used as the enzyme and dissolved in 0.2 mM Tris-HCl buffer (pH 8.0). The substrate, N-Succinyl-Ala-Ala-Ala-p-nitroanilide (SANA), was prepared in the same buffer at a concentration of 1.6 mM. For each reaction, 50 μ L of buffer, 25 μ L of enzyme solution, and 50 μ L of the test extract were pre-incubated at room temperature for 15 min before the addition of SANA. Epigallocatechin gallate (EGCG) was used as the positive control, while ethanol served as the negative control. The reaction was initiated by adding 125 μ L of the substrate solution to the mixture, followed by incubation at room temperature for 20 min. The absorbance change was then measured at 410 nm.

The following formula was used to calculate the percentage of all enzyme inhibitions:

Inhibition (%) = (A_control_–A_sample_)/A_control_ x 100 A: Absorbance.

## 3 Results

### 3.1 Chemical analysis

#### 3.1.1 HPLC-DAD analysis

In the HPLC-DAD analysis, standards utilized included hypericin, pseudohypericin, and hyperforin; however, none of the extracts contained detectable levels of these metabolites (Supplementary Table S1).

#### 3.1.2 LC-HR/MS analysis

In the LC-HR/MS analysis, 26 standards were utilized: ascorbic acid, (−)-epigallocatechin, (−)-epigallocatechin gallate, chlorogenic acid, fumaric acid, (−)-epicatechin, vanillic acid, p-coumaric acid, rutin, hyperoside, dihydrokaempferol, ellagic acid, quercitrin, myricetin, quercetin, salicylic acid, naringenin, kaempferol, 3′-O-methyl quercetin, apigenin, chrysin, emodin, pyrogallol, senecionine N-oxide, hispidulin 7-glucoside, and chrysoeriol. The detailed results are presented in (Supplementary Table S2).

LC-HR/MS analysis revealed a diverse array of bioactive metabolites in the *H. empetrifolium* subsp. *empetrifolium* extracts, with concentrations expressed in μ g/mL. Ascorbic acid was quantified at 52.503 ± 2.069, 35.303 ± 1.391, and 49.879 ± 1.965 μ g/mL in HE-1, HE-2, and HE-3, re-spectively. Among the flavan-3-ols, (−)-epigallocatechin was most abundant in HE-3 (285.744 ± 8.83 μ g/mL), followed by HE-1 (161.37 ± 4.986 μ g/mL) and HE-2 (74.253 ± 2.294 μ g/mL). On the other hand, (−)-epigallocatechin gallate was detected at similar levels across all extracts, with values of 86.167 ± 3.24 μ g/mL for HE-1, 96.588 ± 3.632 μ g/mL for HE-2, and 89.018 ± 3.347 μ g/mL for HE-3.

Chlorogenic acid was present at low levels in HE-1 and HE-3 (approximately 10.960 μ g/mL each) but was markedly lower in HE-2 (2.184 ± 0.078 μ g/mL). Fumaric acid was one of the major constituents, with concentrations of 1,021.486 ± 29.419 μ g/mL in HE-1, 918.493 ± 26.453 μ g/mL in HE-2, and 1,450.265 ± 41.768 μ g/mL in HE-3. Notably, (−)-epicatechin was not detected in HE-1, yet it was observed at 75.881 ± 2.405 μ g/mL in HE-2 and 46.708 ± 1.481 μ g/mL in HE-3.

Vanillic acid was quantified at 264.615 ± 9.235 μ g/mL in HE-1, 119.417 ± 4.168 μ g/mL in HE-2, and 162.998 ± 5.689 μ g/mL in HE-3, while p-coumaric acid was detected in HE-1 (47.567 ± 1.575 μ g/mL) and HE-2 (34.578 ± 1.145 μ g/mL) but was not detected in HE-3. Pyrogallol was detected in HE-1 (1.228 ± 0.055 μ g/mL), HE-2 (1.032 ± 0.046 μ g/mL), and HE-3 (1.009 ± 0.045 μ g/mL). Among the flavonoids, rutin was particularly abundant in HE-1 and HE-2, with levels of 10,009.532 ± 307.293 and 11,444.962 ± 351.36 μ g/mL, respectively, compared to a much lower concentration in HE-3 (648.337 ± 19.904 μ g/mL). In contrast, hyperoside was most concentrated in HE-3 (1,624.24 ± 56.199 μ g/mL) relative to HE-1 (150.715 ± 5.215 μ g/mL) and HE-2 (223.164 ± 7.722 μ g/mL). Quercitrin was detected at low levels in HE-1 (5.84 ± 0.221 μ g/mL) and HE-2 (6.236 ± 236 μ g/mL) but was remarkably high in HE-3 (2,997.251 ± 113.296 μ g/mL). Other flavonoids such as myricetin, quercetin, salicylic acid, and naringenin were present in minor amounts, with quercetin found at around 6.444 ± 0.19–6.638 ± 0.196 μ g/mL across the extracts. Kaempferol and 3′-O-methyl quercetin were either absent or detected at trace levels. Additionally, apigenin, chrysin, and emodin were present in very low concentrations.

Senecionine N-oxide was detected only in HE-1 (0.128 ± 0.005 μ g/mL). Hispidulin 7-glucoside was found in HE-1 (75.892 ± 2.588 μ g/mL) and HE-2 (148.829 ± 5.075 μ g/mL) but was not detected in HE-3, and chrysoeriol was present in both HE-1 (0.152 ± 0.003 μ g/mL) and HE-2 (0.129 ± 0.003 μ g/mL) but absent in HE-3.

### 3.2 Chemical tests assessing radical scavenging capacity

#### 3.2.1 Determination of total phenolic content

Total phenolic contents were quantified as pyrocatechol equivalents (PEs) using the calibration curve y = 0.0307 pyrocatechol ( μ g) + 0.048 (R^2^ = 0.9940). As presented in Supplementary Table S3, extract HE-1 exhibited the highest phenolic concentration at 86.32 ± 1.91 μ g PEs/mg extract, indicating a robust presence of these metabolites. HE-3 also demonstrated a significant phenolic content of 79.80 ± 1.61 μ g PEs/mg extract, whereas HE-2 contained the lowest level at 49.67 ± 1.15 μ g PEs/mg extract.

#### 3.2.2 Determination of total flavonoid content

Total flavonoid content was quantified as quercetin equivalents (QEs) using the calibration curve y = 0.0331 quercetin ( μ g) + 0.0855 (R^2^ = 0.9955). As detailed in Supplementary Table S3, extract HE-3 exhibited the highest flavonoid content at 54.07 ± 0.92 μ g QEs/mg extract, followed by HE-2 with 43.97 ± 0.38 μ g QEs/mg extract. In contrast, HE-1 showed the lowest flavonoid content, measuring 36.81 ± 0.30 μ g QEs/mg extract.

#### 3.2.3 1,1-Diphenyl-2-picrylhydrazyl (DPPH) free radical scavenging assay

The antioxidant capacity of the extracts was assessed using the DPPH (2,2-diphenyl-1-picrylhydrazyl) assay, a widely recognized method for quantifying free radical scavenging capacity. This technique evaluates the ability of the extracts to donate hydrogen atoms or electrons to neutralize DPPH radicals, thereby serving as an indicator of their potential antioxidant efficacy (Gulcin and Alwasel, 2023). At a concentration of 100 μ g/mL, extract HE-1 achieved 82.60 % inhibition, HE-2 reached 79.13 % , and HE-3 showed 77.42 % inhibition. In comparison, the standard antioxidants demonstrated inhibition levels of 75.23 % for BHA, 78.07 % for α -TOC, and 76.17 % for BHT. These results indicate that HE-1 and HE-2, in particular, exhibited strong free radical scavenging capacity, slightly surpassing BHA and BHT, and approaching the efficacy of α -TOC (Supplementary Table S4).

#### 3.2.4 2,2′-azino-bis (3-ethylbenzothiazoline-6-sulfonic acid) (ABTS) cation scavenging assay

The antioxidant capacity of the extracts was evaluated using the ABTS (2,2′-azino-bis (3-ethylbenzothiazoline-6-sulfonic acid)) assay, a well-established method for quantifying free radical scavenging capacity. In this assay, the ABTS radical cation is generated and its reduction by the extracts is measured, providing an indication of their antioxidant efficacy (Nenadis et al., 2004). The corresponding results are detailed in Supplementary Table S4. At 100 μ g/mL, extract HE-1 exhibited the highest scavenging capacity with 88.53 % inhibition, followed by HE-2 at 88.53 % and HE-3 at 88.08 % . In contrast, the reference standards showed inhibition values of 87.62 % for BHA, 88.89 % for α -TOC, and 89.13 % for BHT. These results demonstrate that all three extracts possessed potent ABTS radical scavenging capacity, comparable to or slightly below the capacity of the standard antioxidants.

#### 3.2.5 Cupric reducing antioxidant capacity (CUPRAC) method

The antioxidant capacity of the extracts was further evaluated using the CUPRAC (cupric reducing antioxidant capacity) assay, a well-established method for assessing the electron-donating potential of antioxidants (Özyürek et al., 2011). TThe corresponding results are presented in Supplementary Table S4. At 100 μ g/mL, HE-3 demonstrated the strongest reducing power with an absorbance value of 1.712, followed by HE-1 at 2.103 and HE-2 at 1.877. For comparison, BHA, α -TOC, and BHT exhibited absorbance values of 3.769, 2.460, and 3.559, respectively. These values were recorded at 450 nm, which is the standard detection wavelength for the CUPRAC assay. The results suggest that HE-3 had the highest electron-donating capacity among the extracts, approaching that of the synthetic antioxidants, particularly α -TOC and BHT.

### 3.3 Antimicrobial activity

The antimicrobial activity of *H. empetrifolium* subsp. *empetrifolium* extracts (HE-1, HE-2, and HE-3) was evaluated against a range of microbial strains, including Gram (−) bacteria, Gram (+) bacteria, and yeasts (Supplementary Table S5).

Among Gram (−) bacteria, none of the extracts exhibited activity against *E. coli* ATCC 25922, *K. pneumoniae* ATCC 4352, or *P. mirabilis* ATCC 14153, while HE-3 inhibited *P. aeruginosa* ATCC 27853 at 625 μ g/mL, compared to a MIC of 2.4 μ g/mL for ceftazidime. For Gram (+) bacteria, all extracts were active against *S. aureus* ATCC 29213, with HE-1 showing an MIC of 625 μ g/mL and both HE-2 and HE-3 requiring 1,250 μ g/mL, in contrast to cefuroxime-Na (MIC = 1.2 μ g/mL). Additionally, *E. faecalis* ATCC 29212 was inhibited by all extracts at 1,250 μ g/mL, whereas no activity was observed against *S. epidermidis* ATCC 12228. In the case of yeast strains, all extracts inhibited *C. albicans* ATCC 10231 at 156.2 μ g/mL, while *C. parapsilosis* ATCC 22019 was inhibited at 78.12 μ g/mL by HE-1 and HE-3 (156.2 μ g/mL for HE-2), and *C. tropicalis* ATCC 750 was inhibited at 78.12 μ g/mL across all extracts. These activities are notably less potent than those of the positive controls, with MIC values of 4.9 μ g/mL for cefuroxime-Na, 1.2 μ g/mL for cefuroxime-Na (against *S. aureus*), 128 μ g/mL for amikacin (against *E. faecalis*), 4.9 μ g/mL for clotrimazole (against *C. albicans*), and 1–0.5 μ g/mL for amphotericin B (against *C. parapsilosis* and *C. tropicalis*, respectively).

### 3.4 Enzyme inhibition activity assays

#### 3.4.1 Anti-cholinesterase activity assay

Cholinesterases, such as acetylcholinesterase (AChE) and butyrylcholinesterase (BChE), are essential enzymes responsible for the hydrolysis of acetylcholine, a neuro-transmitter critical for effective synaptic transmission. Inhibition of these enzymes represents a targeted therapeutic strategy in neurodegenerative disorders—particularly Alzheimer’s disease—as it aims to preserve acetylcholine levels and mitigate cognitive decline (Francis et al., 1999; Hampel et al., 2019). This study evaluated the inhibitory potential of *H. empetrifolium* subsp. *empetrifolium* extracts on both AChE and BChE, enzymes that are pivotal for neural communication and constitute significant therapeutic targets for these diseases (Ahmad et al., 2024). The results are presented in Supplementary Table S6.

The HE-1 extract exhibited the strongest inhibitory activity against acetylcholinesterase (AChE), with an IC_50_ value of 8.16 ± 0.39 μ g/mL—closely matching the standard inhibitor Galantamine (IC_50_ = 8.53 ± 0.20 μ g/mL)—while HE-2 and HE-3 demonstrated moderate and weak inhibition (IC_50_ = 17.55 ± 0.82 μ g/mL and 42.09 ± 1.48 μ g/mL, respectively).

Moreover, the butyrylcholinesterase (BChE) inhibition assays demonstrated that HE-1 exhibited the strongest activity, with an IC_50_ value of 2.46 ± 0.02 μ g/mL, significantly better than Galantamine (IC_50_ = 38.66 ± 0.49 μ g/mL). In comparison, HE-2 and HE-3 had IC_50_ values of 13.46 ± 0.42 μ g/mL and 26.42 ± 0.86 μ g/mL, respectively.

#### 3.4.2 Anti-tyrosinase activity assay

This study evaluated the inhibitory effects of *H. empetrifolium* subsp. *empetrifolium* extracts on tyrosinase, a key enzyme in melanin biosynthesis and pigment production. Tyrosinase inhibitors are essential for addressing hyperpigmentation and are extensively studied for their potential in cosmetic applications (Samaneh et al., 2019). The IC_50_ values, representing the concentration of extracts required to inhibit 50 % of tyrosinase activity, were determined and are presented in Supplementary Table S6. The extracts exhibited moderate inhibitory activity, with IC_50_ values of 110.02 ± 0.91 μ g/mL, 101.76 ± 0.35 μ g/mL, and 98.56 ± 1.31 μ g/mL, respectively. In comparison, the standard inhibitor kojic acid demonstrated a significantly lower IC_50_ value of 21.70 ± 0.97 μ g/mL. Although less potent than kojic acid.

#### 3.4.3 Anti- α -glucosidase activity assay

This study investigated the inhibitory potential of *H. empetrifolium* subsp. *empetrifolium* extracts on α -Glucosidase, a key enzyme involved in carbohydrate digestion and glucose regulation, making it a therapeutic target for diabetes management (Dirir et al., 2022a). The results indicate remarkable potence with IC_50_ values of 26.2 ± 1.52 μ g/mL, 30.27 ± 1.04 μ g/mL, and 30.91 ± 0.51 μ g/mL, respectively. In contrast, the standard inhibitor acarbose, a well-known α -Glucosidase inhibitor, exhibited a significantly higher IC_50_ value of 676.5 ± 10.5 μ g/mL. These findings highlight the exceptional inhibitory potential of HE extracts, demonstrating a substantially stronger effect than acarbose. The results are presented in Supplementary Table S6.

#### 3.4.4 Anti-aging activity assay

##### 3.4.4.1 Anti-hyaluronidase activity assay

This study evaluated the inhibitory potential of *H. empetrifolium* subsp. *empetrifolium* extracts on hyaluronidase, an enzyme responsible for degrading hyaluronic acid—a key constituent of the skin’s extracellular matrix that maintains hydration and structural integrity. Given the role of hyaluronidase in skin aging and inflammatory processes, its inhibition represents a promising strategy in anti-aging, and dermatological applications (Jung, 2020). The *in vitro* hyaluronidase inhibitory activity of HE-1, HE-2, and HE-3 extracts could not be determined, indicating no measurable inhibition under the tested conditions. In contrast, the reference inhibitor ursolic acid exhibited a significant inhibitory effect, with an IC_50_ value of 78.62 ± 1.46 μ g/mL. The results are presented in Supplementary Table S6.

##### 3.4.4.2 Anti-elastase activity assay

This study assessed the inhibitory potential of *H. empetrifolium* subsp. *empetrifolium* extracts on elastase, a critical enzyme involved in elastin degradation—a key factor in maintaining skin elasticity. Given elastase’s role in skin aging and connective tissue deterioration, its inhibition is a promising strategy in anti-aging and dermatological applications (Pitasi et al., 2024). *In vitro* assays revealed that extracts HE-1, HE-2, and HE-3 exhibited IC_50_ values of 17.04 ± 0.18 μ g/mL, 17.12 ± 0.14 μ g/mL, and 21.79 ± 0.84 μ g/mL, respectively. In comparison, the reference inhibitor, ursolic acid, demonstrated a lower IC_50_ value of 13.77 ± 0.17 μ g/mL, indicating that while the HE extracts show moderate elastase inhibition, they are slightly less potent than ursolic acid. The detailed results are presented in Supplementary Table S6.

## 4 Discussion

Fungal infections represent a major public health challenge, with mortality rates comparable to malaria, tuberculosis, or HIV (Gow et al., 2022). Recent epidemiological studies have documented a rising incidence of candidemia in ICU settings, underscoring the need for novel antifungal therapies (Eun et al., 2020; Zhong et al., 2022). *Hypericum* species have attracted attention due to their antimicrobial properties, particularly against Gram (+) bacteria, and certain species have demonstrated significant antifungal properties certain fungal pathogens (Tavlı, 2024).

For example, among seven *Hypericum* species from the And Mountains, *Hypericum garciae* exhibited outstanding antifungal activity against *Candida* strains; its methanol extract achieved ${\text{MIC}}_{50}$ values of 5.04 μ g/mL against *C. albicans* and 4 μ g/mL against *C. lusitaniae*, while its chloroform extract outperformed fluconazole against *C. tropicalis* ( ${\text{MIC}}_{50}$ = 39.19 μ g/mL), likely reflecting its high content of quercetin-3-glucuronide, procyanidin B2, and epicatechin (Tocci et al., 2018b). Similarly, *Hypericum hircinum* subsp. *majus* extracts—prepared in methanol, 80 % ethanol, and water—demonstrated significant activity against both fluconazole-sensitive and -resistant *Candida* strains, with the methanol extract showing an ${\text{MIC}}_{50}$ of 53.5 μ g/mL against *C. parapsilosis* and notable effects against *C. tropicalis, C. glabrata, C. albicans*, and *C. lusitaniae* (Tocci et al., 2018a).

Synergistic effects have also been reported; the lipophilic fraction of *Hypericum carinatum* from Brazil, when combined with fluconazole, reduced MIC values by up to eight-fold against *C. krusei* and *C. famata*, although fluconazole alone was more effective against *C. parapsilosis* and *C. neoformans* (Meirelles et al., 2017). Additionally, n-hexane extracts from five *Hypericum* species collected in Brazil showed potent antifungal effects against opportunistic yeasts such as *Cryptococcus neoformans* (MIC ≤ 15.6 μ g/mL) and *Rhodotorula mucilaginosa* (MIC ≤ 62.5 μ g/mL), with *Hypericum myrianthum* notably rich in dimeric phloroglucinol derivatives like uliginosin B and japonisin A (Barros et al., 2013).

Methanol extracts from *Hypericum humifusum* and *Hypericum perfoliatum*, collected in Tunisia, were effective against *C. albicans* (MIC = 250 μ g/mL), with *H. humifusum* containing significant levels of hypericin (90 mg/g) and hyperforin (30 mg/g) (Bejaoui et al., 2017). In addition, 50 % ethanol extracts from the leaves and roots of *Hypericum havvae* demonstrated strong antifungal activity against several yeast strains, particularly when combined, achieving MIC values as low as 1.56 mg/mL against *C. albicans* and *C. laurentii* (Dulger and Dulger, 2016). Lastly, methanol extracts from six *Hypericum* species cultivated in Türkiye confirmed antifungal efficacy, with diethyl ether and chloroform extracts of *Hypericum spectabile* showing activity against *C. albicans* ATCC 10231 (MIC = 156 μ g/mL) (Ero et al., 2018b).

**FIGURE 1:**
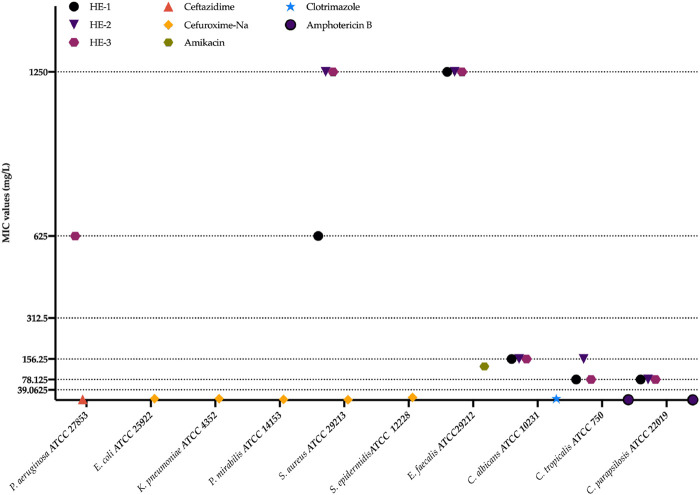
Antimicrobial activity assays. HE-1,2,3: extract codes. Ceftazidime, Cefuroxime-Na, Amikacin, Clotrimazole, and Amphotericin B: standard compounds.

Our previous results indicated that the ethanolic extract obtained from the aerial parts of *H. empetrifolium* subsp. *empetrifolium* exhibited significant antifungal activity, with MIC values of 4.88 μ g/mL against *C. parapsilosis*, 19.53 μ g/mL against *C. tropicalis*, and 78.12 μ g/mL against *C. albicans* (Bo et al., 2021). In the present study, methanolic extracts prepared from *H. empetrifolium* subsp. *empetrifolium* samples collected from different geographical locations also exhibited anti-fungal activity against the same heast strains with the IC_50_ values ranging from 78.12 to 156.2 μ g/mL. Their effectiveness was comparable to standard antifungal agents used in clinical practice. However, the extracts showed no significant activity against Gram (−) bacteria, which is consistent with existing literature. Interestingly, weak inhibitory activity was observed against the tested Gram (+) bacterial strains (Figure 1).

These results were not as robust as those observed in our previous study. This discrepancy may be attributed to extractant solvent differences or variations in the chemical composition of the plants, which can be influenced by differences in their growth conditions.

Although both ethanol and methanol are polar solvents commonly used in phytochemical research, their slight differences in polarity and solvent strength can lead to distinct extraction efficiencies for certain classes of secondary metabolites. Methanol, being slightly more polar than ethanol, may enhance the extraction of certain phenolic acids and low-molecular-weight flavonoids, whereas ethanol might favor more lipophilic or mid-polar metabolites (Chaves et al., 2020). In a comparative study on *Hypericum perforatum*, Alahmad et al. (2022) observed that while the overall compound profiles were similar across different solvents, water tended to yield higher concentrations of certain phenolic constituents compared to methanol and ethanol (Alahmad et al., 2022). On the other hand, various phloroglucinol derivatives, which have low water solubility, are known for their potent antimicrobial properties (Tavlı, 2024). This suggests that methanol may offer advantages in extracting specific bioactive metabolites, owing to its ability to extract both phenolics and phloroglucinol derivatives effectively.

This variation in solvent polarity likely contributed to the observed differences in antifungal activity between the two studies. Additionally, the chemical composition of the extracts may have been influenced not only by the choice of solvent, but also by ecological and geographical differences in the plant material, including factors such as altitude, climate, and soil conditions. These environmental variables are known to affect the biosynthesis and accumulation of bioactive metabolites in medicinal plants and could therefore play a significant role in the observed phytochemical variation and corresponding biological activities (Çirak et al., 2006; Çirak and Radusiene, 2019; Şenkal and Uskutoglu, 2021; Bálintová et al., 2019; Kurt et al., 2018; Kucharíková et al., 2016; Kladar et al., 2015; Ghasemi Pirbalouti et al., 2011; Hosni et al., 2011; Çırak et al., 2011; Maggi et al., 2010; AI-Rifaee et al., 2010; Toker, 2009; Bagdonaite et al., 2009; Çirak et al., 2008; Mártonfi et al., 2006; Božin et al., 2013).

Phytochemical analysis revealed that the ethanolic extract of *H. empetrifolium* subsp. *empetrifolium* was particularly rich in chlorogenic acid, isoquercitrin, malic acid, proto-catechuic acid, quercetin, and fumaric acid. Additionally, several other phenolic metabolites—including salicylic acid, caffeic acid, p-coumaric acid, rutin, nicotiflorin, rosmarinic acid, naringenin, apigenin, and vanillin—were identified at lower concentrations.

Similarly, chemical examination of the *H. empetrifolium* subsp. *empetrifolium* extracts revealed marked differences in their phytochemical profiles. For instance, while ascorbic acid levels were relatively similar among the samples notable variations were observed for several key flavonoids and phenolic acids. HE-3 exhibited a substantially higher concentration of (−)-epigallocatechin (285.744 ± 8.83 μ g/mL) compared to HE-1 (161.37 ± 4.986 μ g/mL) and HE-2 (74.253 ± 2.294 μ g/mL), suggesting a stronger potential for antioxidant effect. Similarly, hyperoside was present in much higher amounts in HE-3 (1.624.24 ± 56.199 μ g/mL) relative to HE-1 (150.715 ± 5.215 μ g/mL) and HE-2 (223.164 ± 7.722 μ g/mL), whereas rutin levels were considerably higher in HE-1 and HE-2 (10.009.532 ± 307.293, 11.444.962 ± 351.36 μ g/mL, respectively) than in HE-3 (648.337 ± 19.904 μ g/mL). In contrast, quercitrin was detected at an exceptionally high level in HE-3 (2.997.251 ± 113.296 μ g/mL) compared to low concentrations in HE-1 and HE-2 (5.84 ± 0.221, 6.236 ± 236 μ g/mL, respectively). Other metabolites, such as chlorogenic acid and fumaric acid, displayed moderate variability across the extracts, while some metabolites like dihydrokaempferol and certain flavonoids (e.g., chrysin, 3′-O-methyl quercetin) were either absent or present only in trace amounts in specific samples.

Reactive oxygen species (ROS), including free radicals such as superoxide ( ${\text{O}}_{2}•$ ), hydroxyl (HO • ), peroxyl (LOO • ), hydroperoxyl (HOO • ), and non-radical molecules like hydrogen peroxide ( ${\text{H}}_{2}{\text{O}}_{2}$ ), are generated as by-products of mitochondrial respiration, enzymatic reactions, and exposure to both internal and external factors such as inflammation, exercise, ischemia-reperfusion injury, metal ions, respiratory burst, cigarette smoke, and industrial solvents (Foret et al., 2020; Neha et al., 2019). Although cells continuously produce ROS as signaling molecules involved in immune response, cell signaling, wound healing, and pathogen defense (Pandey et al., 2021; Schieber and Navdeep, 2014; Sies, 2018), excessive accumulation or inadequate antioxidant defense systems lead to oxidative stress—a key contributor to the pathogenesis of various diseases including cancer, cardiovascular diseases, neurodegenerative disorders, diabetes, rheumatoid arthritis, kidney, and ocular diseases (Foret et al., 2020; Pisoschi et al., 2021; Saddiqe et al., 2014; Pandey et al., 2021). ROS-induced oxidative damage to proteins, lipids, and nucleic acids may result in cell death, inflammation, LDL oxidation, or impaired insulin signaling, depending on the disease context. Plants have evolved complex antioxidant defense systems, including enzymatic and non-enzymatic metabolites such as ascorbic acid, glutathione, polyphenols, flavonoids, and terpenes (Llauradó Maury et al., 2020). While synthetic antioxidants like BHA, BHT, and propyl gallate have been widely used, their long-term safety has raised concerns due to associations with allergies, gastrointestinal issues, carcinogenic potential, and DNA damage in high doses (Lourenço et al., 2019). Consequently, there is a growing interest in plant-derived antioxidants as safer alternatives for applications in food, pharmaceuticals, and cosmetics (Pammi et al., 2022).

**FIGURE 2:**
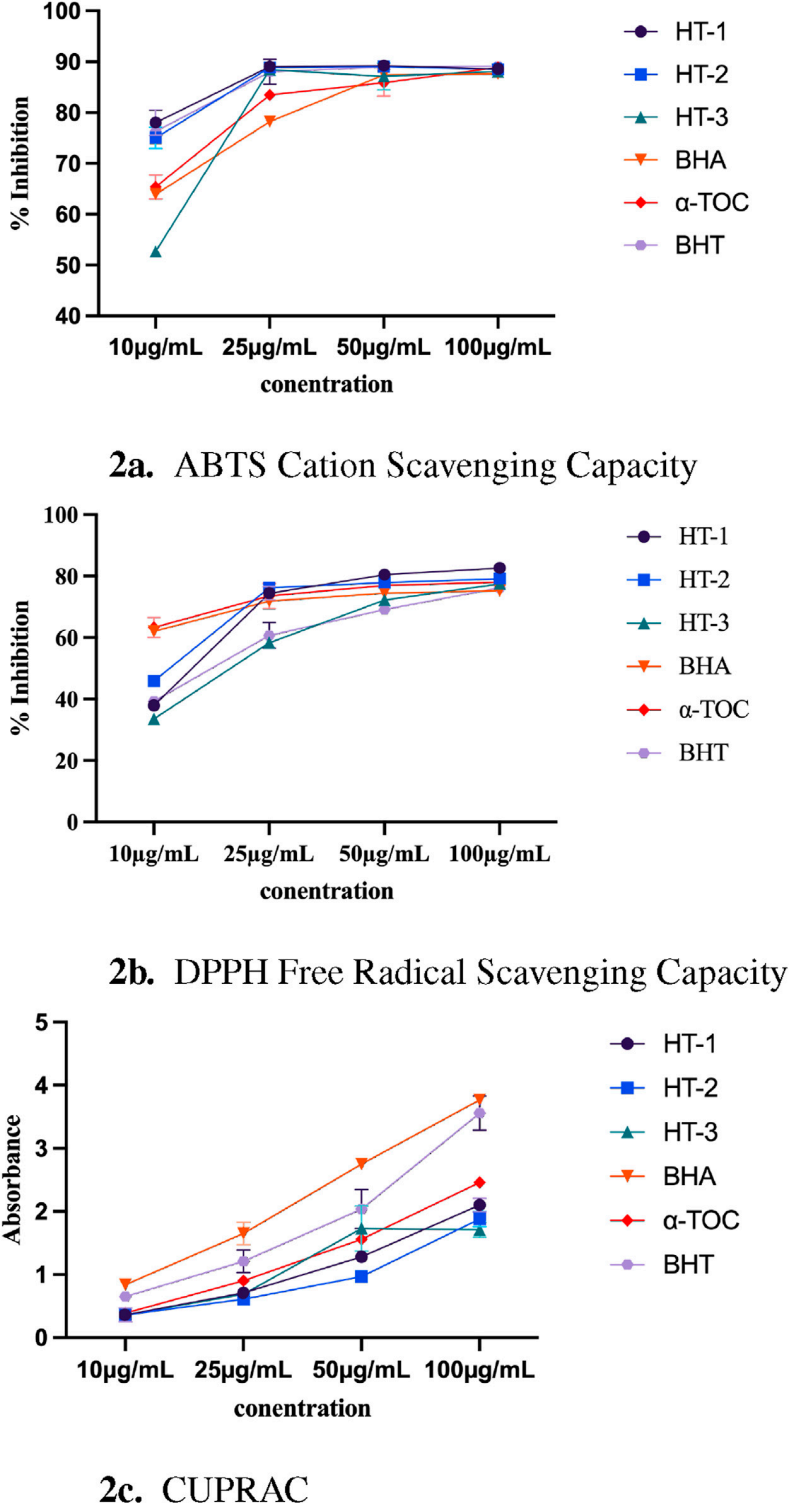
**(a)** ABTS Cation Scavenging Capacity. **(b)** DPPH Free Radical Scavenging Capacity. **(c)** CUPRAC. Chemical tests assessing radical scavenging capacity results. HE-1,2,3: extract codes. BHA, α -TOC, and BHT: standard compounds Statistical significance levels are indicated as follows: (^**^) P≤0.01 ; (^***^) P≤0.001 ; (^****^) P≤0.0001 .

The radical scavenging capacities of the extracts were assessed using three distinct chemical methods.The results were as follows: maximum inhibition values of 82.60 % in the DPPH assay, 88.53 % in the ABTS assay, and an absorbance of 2.103 in the CUPRAC assay, all determined at a concentration of 100 μ g/mL. Notably, HE-1 showed the highest radical scavenging capacity in the ABTS assay, clearly outperforming the other samples. In contrast, HE-2 displayed slightly better free radical scavenging in the DPPH assay (82.60 % at 100 μ g/mL) compared to HE-1, while HE-3 demonstrated the most potent reducing power in the CUPRAC assay (Abs = 1.712 at 100 μ g/mL) (Figure 2). These differences likely reflect the variations in their phenolic and flavonoid contents, underlining the importance of phytochemical composition in determining antioxidant efficacy. It is important to note that these results are based solely on chemical assays and do not imply any pharmacological efficacy. Such methods are valuable analytical tools for characterizing antioxidant potential but cannot be equated with biological activity. Therefore, the findings should be interpreted as indicators of radical scavenging capacity rather than evidence of *in vitro* or *in vivo* antioxidant effects.

To date, different investigations in literature have not only highlighted differences among species but also revealed variations in the antioxidant potential of the same species (particularly *H. perforatum*) sourced from different regions. Among of them, a study showed that among three *Hypericum* species grown in Türkiye—*Hypericum aviculariifolium* subsp. *depilatum* var. *depilatum*, *Hypericum salsugineum*, and H*. perforatum*—the methanolic extracts exhibited DPPH radical scavenging capacity of 88.29 % , 86.88 % , and 81.21 % , respectively, at a concentration of 0.5 mg/mL (Maltaş et al., 2013). In the Balkans, *H. perforatum* has been reported to possess a DPPH free radical scavenging capacity with IC_50_ value of 1.36 ± 0.05 μ g/mL (Božin et al., 2013), while in Greece, *H. perforatum* (DPPH; IC_50_ = 10.45 ± 0.61 μ g/mL), *Hypericum delphicum* (DPPH; IC_50_ = 12.98 ± 1.09 μ g/mL), and *Hypericum olympicum* (ABTS; IC_50_ = 3.92 ± 0.73 μ g/mL) have demonstrated notable antioxidant effects (Kakouri et al., 2023). Moreover, a study from Bulgaria evaluating the antioxidant effects of *H. olympicum* and *H. perforatum* found that *H. perforatum*, which had the highest total tannin content (8.67 ± 0.02 g pyrogallol equivalents/100 g), exhibited radical scavenging capacities of 77.6 ± 0.5 % in the DPPH assay and 81.2 ± 0.4 % in the ABTS assay. In contrast, *H. olympicum*, despite its lower total flavonoid content (0.20 ± 0.03 g hyperoside equivalents/100 g), showed a strong total antioxidant effect of 89.9 ± 0.2 μ M Trolox equivalents/g (Zheleva-Dimitrova et al., 2010). The chemical tests assessing radical scavenging capacity results obtained in the present study are in agreement with these literature findings.

**FIGURE 3:**
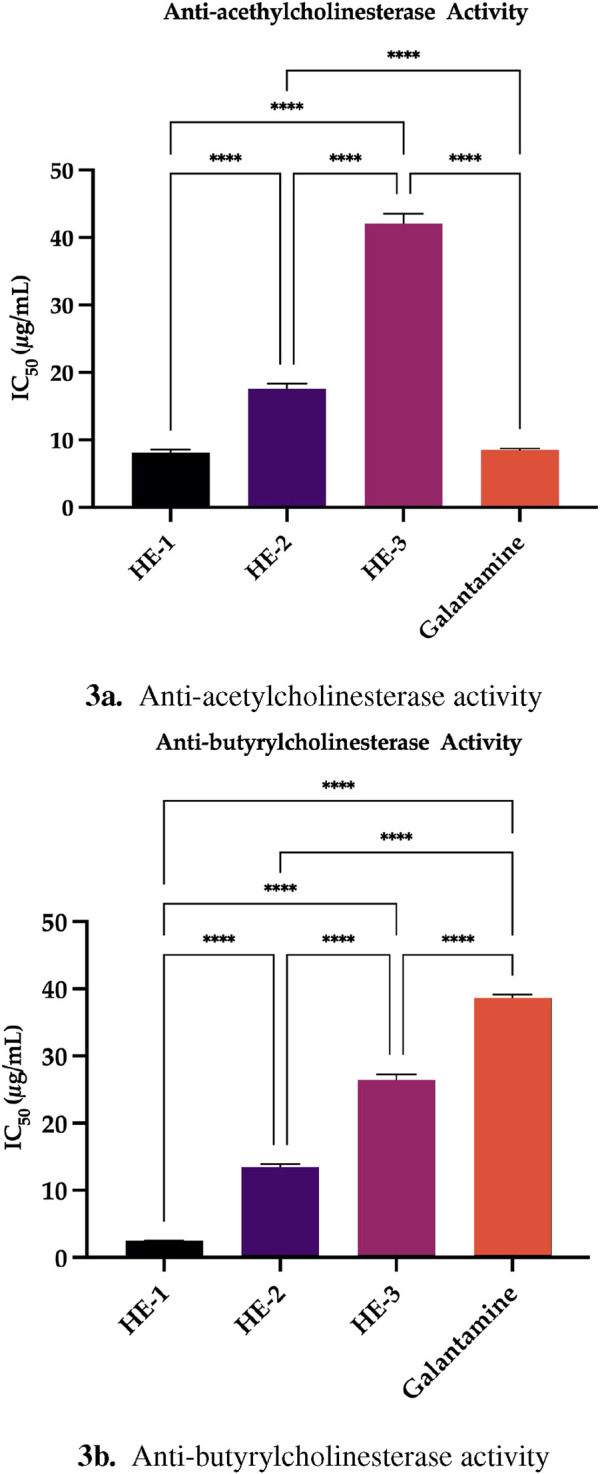
**(a)** Anti-acetylcholinesterase activity. **(b)** Anti-butyrylcholinesterase activity. Anti-cholinesterase activity results. HE-1,2,3: extract codes. Galantamine, kojic acid, acarbose, and ursolic acid: standard compounds. Statistical significance levels are indicated as follows: (^*^) P≤0.05 ; (^**^) P≤0.01 ; (^***^) P≤0.001 ; (^****^) P≤0.0001 .

Studies on cholinesterase inhibitors have demonstrated that, beyond their established efficacy in Alzheimer’s disease, these agents may also exert potential effects on mood disorders related to depression and stress. Consequently, cholinesterase inhibitors are being considered for their potential role in the treatment of depression, in addition to neurodegenerative conditions (Fitzgerald et al., 2020). Based on the anticholinesterase activity assays results, *H. empetrifolium* subsp. *empetrifolium* extracts—especially HE-1—demonstrate promising potential for the treatment of these conditions. In the present study, the cholinesterase inhibitory activities of the extracts were determined as IC_50_ values ranging from 8.16 ± 0.39 to 42.09 ± 1.48 μ g/mL for acetylcholinesterase (AChE) and from 2.46 ± 0.02 to 26.42 ± 0.86 μ g/mL for butyrylcholinesterase (BChE) (Figure 3). Notably, HE-1 exhibited the most potent inhibitory effects with an AChE IC_50_ of 8.16 ± 0.39 μ g/mL and a BChE IC_50_ of 2.46 ± 0.02 μ g/mL, outperforming the standard galantamine in BChE inhibition (galantamine: AChE, 8.53 ± 0.20 μ g/mL; BChE, 38.66 ± 0.49 μ g/mL). In contrast, HE-2 and HE-3 demonstrated relatively weaker activities. Not only present results but also our previous findings (Methanolic extract of *H. empetrifolium* subsp. *empetrifolium* inhibited 38.89 ± 1.07 % AChE, and 88.69 ±  0.62 % BChE at 200 μ g/mL concentration (Bo et al., 2021)) suggesting that variations in their phytochemical profiles may significantly influence their cholinesterase inhibitory potential.

On the other hands, various studies have explored the cholinesterase inhibitory effects of *Hypericum* species, shedding light on their potential as natural anticholinesterase agents. Bozin and colleagues evaluated the anti-acetylcholinesterase activity of extracts of *H. perforatum*, *Hypericum maculatum* subsp. *immaculatum*, *H. olympicum*, *Hypericum richeri* subsp. *grise-bachii*, and *Hypericum barbatum*, reporting that the *H. perforatum* extract exhibited the highest activity (IC_50_ = 432.74 μ g/mL), which was attributed to its high hyperforin content (Božin et al., 2013). In another study demonstrated that methanolic extracts prepared from the aerial parts of *Hypericum neurocalycinum* and *Hypericum malatyanum* possessed notable anti-acetylcholinesterase effects, with EC_50_ values of 2.16 mg/mL and 6.83 mg/mL, respectively, suggesting that flavonoids such as quercetin, kaempferol, and rutin might be responsible for this activity (Ero et al., 2018a). Furthermore, Ersoy and coworkers investigated the anti-cholinesterase activities of methanolic extracts from *Hypericum calycinum*, *Hypericum confertum*, and *H. perforatum* at a concentration of 200 μ g/mL, finding that *H. calycinum* exhibited the highest acetylcholinesterase inhibition (45.33 % ), while *H. perforatum* showed the strongest butyrylcholinesterase inhibition (82.50 % ). These extracts were rich in phenolic metabolites including chlorogenic acid, hyperoside, quercetin, rutin, and isoquercitrin (Ersoy et al., 2019). Additionally, a decoction prepared from *Hypericum androsaemum*, *Hypericum undulatum*, and *H. perforatum* displayed potent anti-acetylcholinesterase activity, with IC_50_ values ranging from 0.62 ± 0.06 to 1.79 ± 0.37 μ g/mL. The major constituents of these extracts—chlorogenic acid, rutin, hyperoside, isoquercitrin, and quercetin—exhibited individual IC_50_ values between 196 and 62 μ g/mL (Hernandez et al., 2010), further supporting the hypothesis that the anti-cholinesterase effects of *Hypericum* extracts result from the synergistic interactions among their bioactive metabolites.

The investigation of tyrosinase enzyme inhibitors is of considerable importance for the discovery of novel therapeutic agents targeting a variety of health issues. These inhibitors effectively suppress the activity of tyrosinase—an enzyme critical for melanin synthesis—thereby offering potential benefits against hyperpigmentation and skin discoloration. In the cosmetic industry, such compounds are widely incorporated into skin whitening and spot treatment products, as they play a pivotal role in reducing melanin production and treating hyperpigmentation disorders. Moreover, the oxidative stress-related effects associated with tyrosinase are currently under investigation for their implications in neurodegenerative diseases, particularly in conditions such as Parkinson’s disease (Baber et al., 2023; Fernandes and Kerkar, 2017). Previous studies have demonstrated that extracts from various *Hypericum* species exhibit significant antityrosinase activity. An extract prepared from *H. androsaemum* fruits—rich in phenolic metabolites such as shikimic acid and chlorogenic acid—showed notable tyrosinase inhibition (IC_50_ = 229,1 μ g/mL) (López et al., 2016). Similarly, an extract obtained from the aerial parts of *H. calycinum*, enriched in chlorogenic acid, quercitrin, quinic acid, and isoquercitrin, also displayed potent antityrosinase activity (54,30 ± 0,49 % in 200 μ g/mL concentration) (Ersoy et al., 2020). Moreover, an extract of *Hypericum laricifolium* containing protocatechuic acid, p-hydroxybenzoic acid, chlorogenic acid, vanillic acid, caffeic acid, kaempferol 3-O-glucuronide, quercetin, and kaempferol was reported to possess high antityrosinase effects (GuillenQuispe et al., 2017). Additionally, numerous metabolites have been evaluated for their tyrosinase inhibitory activity, with studies indicating that both simple phenolic metabolites and their polyphenolic derivatives exhibit strong antityrosinase activity (S et al., 2019).

**FIGURE 4:**
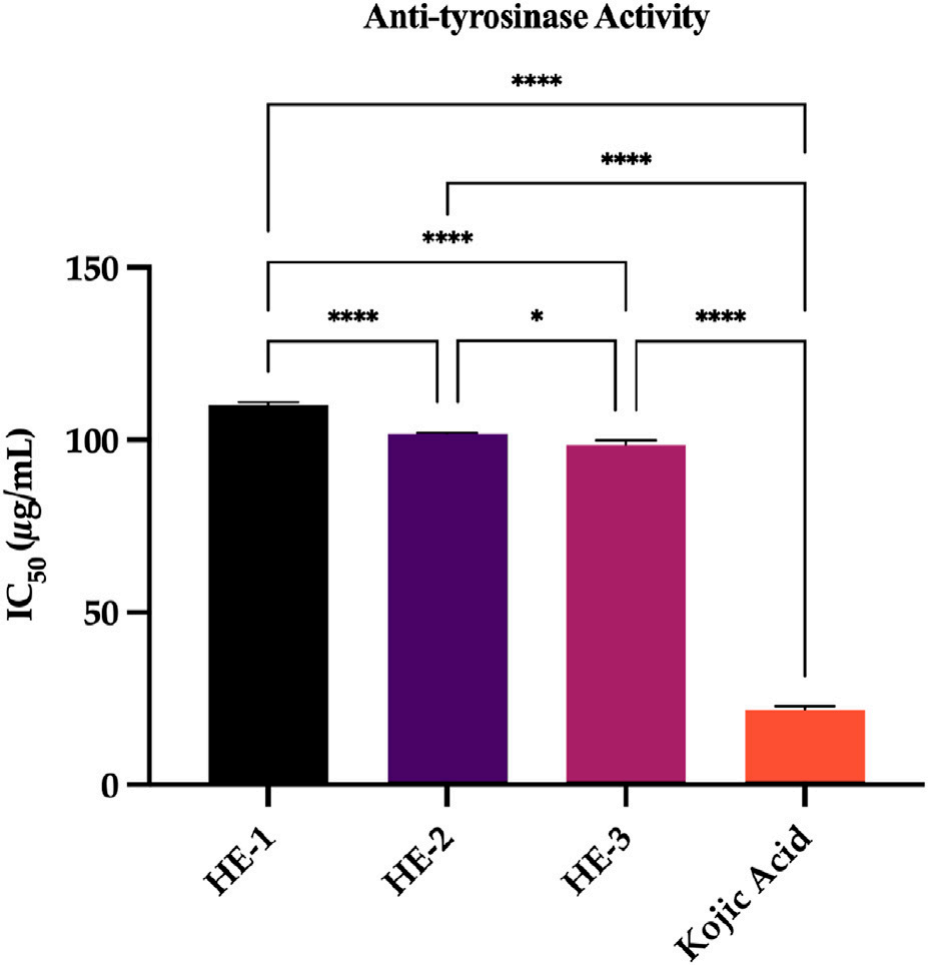
Anti-tyrosinase activity. Kojic acid: standard compound. Statistical significance levels are indicated as follows: (^**^) P≤0.05 ; (^***^) P≤0.001 ; (^****^) P≤0.0001 .

In the present study, the tyrosinase inhibitory potential of *H. empetrifolium* subsp. *empetrifolium* extracts was further evaluated. These findings suggest that although *H. empetrifolium*extracts possess antityrosinase activity, their inhibitory potency is substantially lower than that of kojic acid (Figure 4), similar to previous work (Bo et al., 2021).

α -Glucosidase inhibitors play a critical role in the management of type 2 diabetes, particularly in controlling postprandial blood glucose levels. These inhibitors slow the conversion of complex carbohydrates into simple sugars, thereby delaying glucose absorption and contributing to more stable blood sugar levels. Consequently, they hold significant potential for reducing the long-term complications associated with diabetes (Dirir et al., 2022b).

*H.ypericumascyron* extracts prepared with ethyl acetate and methanol inhibited α -Glucosidase with IC_50_ values of 755.8 μ g/mL and 151.47 μ g/mL, respectively, while isolated constituents such as kaempferol and ursolic acid were particularly potent, with ursolic acid exhibiting an IC_50_ of 1.78 μ M and kaempferol showing values of 61.83 μ M (Kang et al., 2011). In addition, studies on *Hypericum attenuatum* have demonstrated significant antidiabetic effects both *in vitro* and *in vivo*. A phenolic-rich extract improved hyperglycemia, dyslipidemia, and insulin resistance in KK-Ay mice, with observed benefits on hepatic steatosis and preservation of pancreatic β -cells. Moreover, an ethanol extract of *H. attenuatum* yielded isolated flavonoids—such as astragalin, guaijaverin, and quercetin—with ${\text{IC}}_{50}$ values of 33.90 ± 0.68 μ M, 17.23 ± 0.75 μ M, and 31.90 ± 0.34 μ M, respectively. These metabolites not only directly inhibited α -Glucosidase but also induced conformational changes in the enzyme, as demonstrated by circular dichroism and molecular docking studies, with certain combinations displaying synergistic inhibitory effects (Jin et al., 2021). Further supporting these findings, *H. laricifolium* methanol extracts exhibited strong α -Glucosidase inhibition ( ${\text{IC}}_{50}$ = 56.6 μ g/mL) alongside significant aldo reductase and antioxidant capacities. In this extract, quercetin and kaempferol were identified as major contributors, with quercetin showing an ${\text{IC}}_{50}$ value of 15.9 μ M for α -Glucosidase and kaempferol at 9.7 μ M (YN et al., 2017). Similarly, *H. olympicum*, after *in vitro* digestion simulation, demonstrated enhanced inhibition of both α -Glucosidase and α -amylase, with maximum inhibitory activities of 40.28 % and 89.11 % , respectively, and a notable suppression of advanced glycation end products (AGEs) formation (Akyuz et al., 2021). Collectively, these studies underscore the promise of *Hypericum* species as sources of α -Glucosidase inhibitors. Their diverse bioactive metabolites, particularly phenolics and flavonoids, not only contribute to direct enzyme inhibition but also may synergistically improve metabolic profiles, paving the way for the development of novel antidiabetic therapies.

**FIGURE 5:**
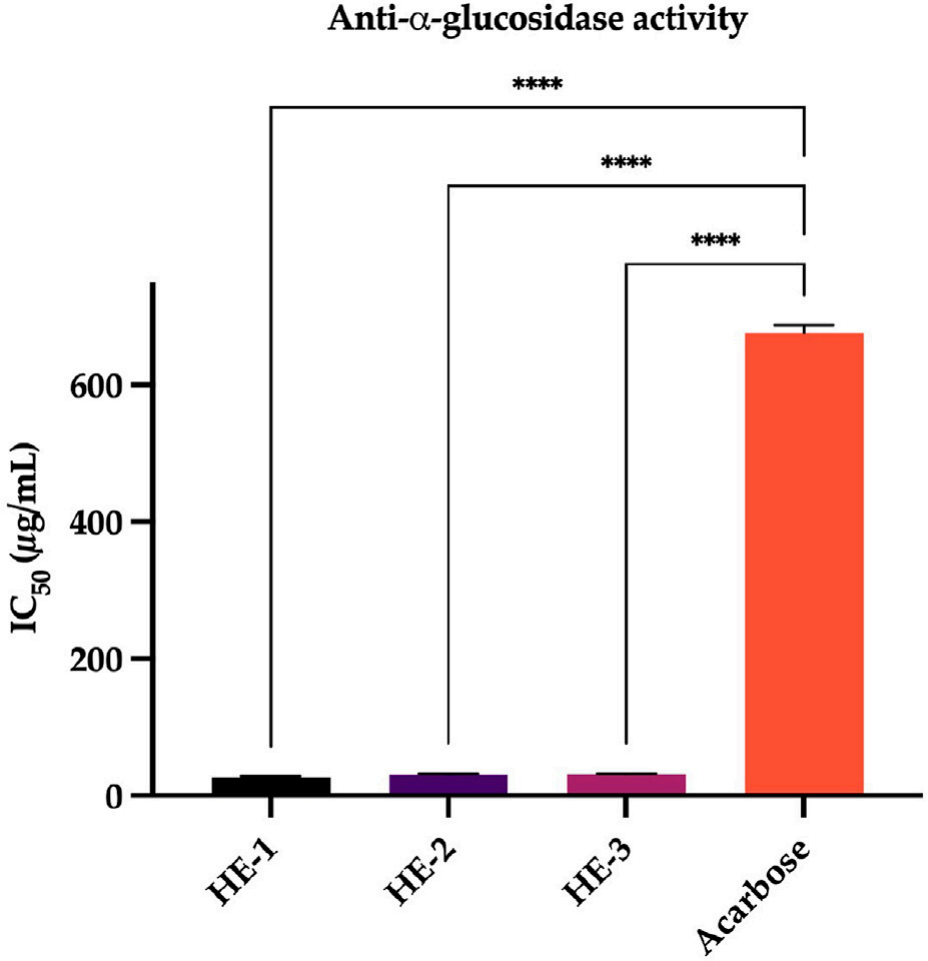
Anti- α -Glucosidase activity. Galantamine: standard compound. Statistical significance levels are indicated as follows: (^****^) P≤0.0001 .

Our findings are particularly noteworthy when compared with the existing literature, where various *Hypericum* species have also been reported to exhibit α -Glucosidase inhibition, albeit with less pronounced potency. The exceptional activity observed in our study suggests that the bioactive constituents within *H. empetrifolium* subsp. *empetrifolium* may offer significant therapeutic potential as novel antidiabetic agents (Figure 5). Given that acarbose is widely used in clinical settings yet has certain limitations, the potent inhibitory effects of these extracts highlight their promise as alternative or complementary treatments with the possibility of improved efficacy and reduced side effects.

Investigating inhibitors of extracellular matrix (ECM) degrading enzymes—such as collagenase, elastase, and hyaluronidase—is critical for developing effective anti-aging cosmeceuticals. These enzymes play a pivotal role in ECM remodeling; their overactivity contributes to the degradation of collagen, elastin, and hyaluronic acid, leading to loss of skin firmness, increased wrinkle formation, and other visible signs of aging. By targeting these enzymes, natural compounds can help preserve the skin’ structural integrity and mitigate age-related changes (Silva et al., 2021).

Studies on various *Hypericum* species have demonstrated promising inhibitory effects on these ECM-degrading enzymes, suggesting that their bioactive constituents could serve as safer and more effective alternatives to synthetic inhibitors in cosmetic formulations. This line of research is therefore essential not only for understanding the mechanisms behind skin aging but also for the development of innovative treatments that improve skin health and appearance. In studies assessing the anti-aging properties of *Hypericum* extracts from *H. perforatum, H. calycinum*, and *H. confertum*. *H. calycinum* consistently demonstrated the most potent activity. Its methanol extracts inhibited collagenase, elastase, and hyaluronidase with ${\text{IC}}_{50}$ values of 51.24, 55.77, and 22.17 μ g/mL, respectively. LC-MS/MS analysis revealed that *H. calycinum* was richer in key bioactive metabolites—such as chlorogenic acid, quercitrin, quinic acid, and isoquercitrin—potentially explaining its superior performance (Ersoy et al., 2019). In addition, *H. androsaemum* fruit extracts promoted fibroblast migration, non-competitively inhibited collagenase, and modulated IL-6 production in PBMCs, indicating promising applications in skin care (Antognoni et al., 2017). Extracts from *H. hircinum, Hypericum origanifolium,* and *Hypericum lydium* further exhibited notable elastase and collagenase inhibition (Mandrone et al., 2015). Together, these findings underscore the potential of *Hypericum* extracts, particularly from *H. calycinum*, as effective agents in combating skin aging, hyperpigmentation, and promoting tissue repair.

The results of present study offer valuable insights into the enzyme inhibitory properties of *H. empetrifolium* subsp. *empetrifolium* extracts. Notably, no measurable inhibition of hyaluronidase was observed for HE-1, HE-2, and HE-3 under the tested conditions. This suggests that the bioactive constituents in the HE extracts may lack the necessary affinity or concentration to effectively inhibit hyaluronidase, an enzyme critical for hyaluronic acid degradation.

**FIGURE 6:**
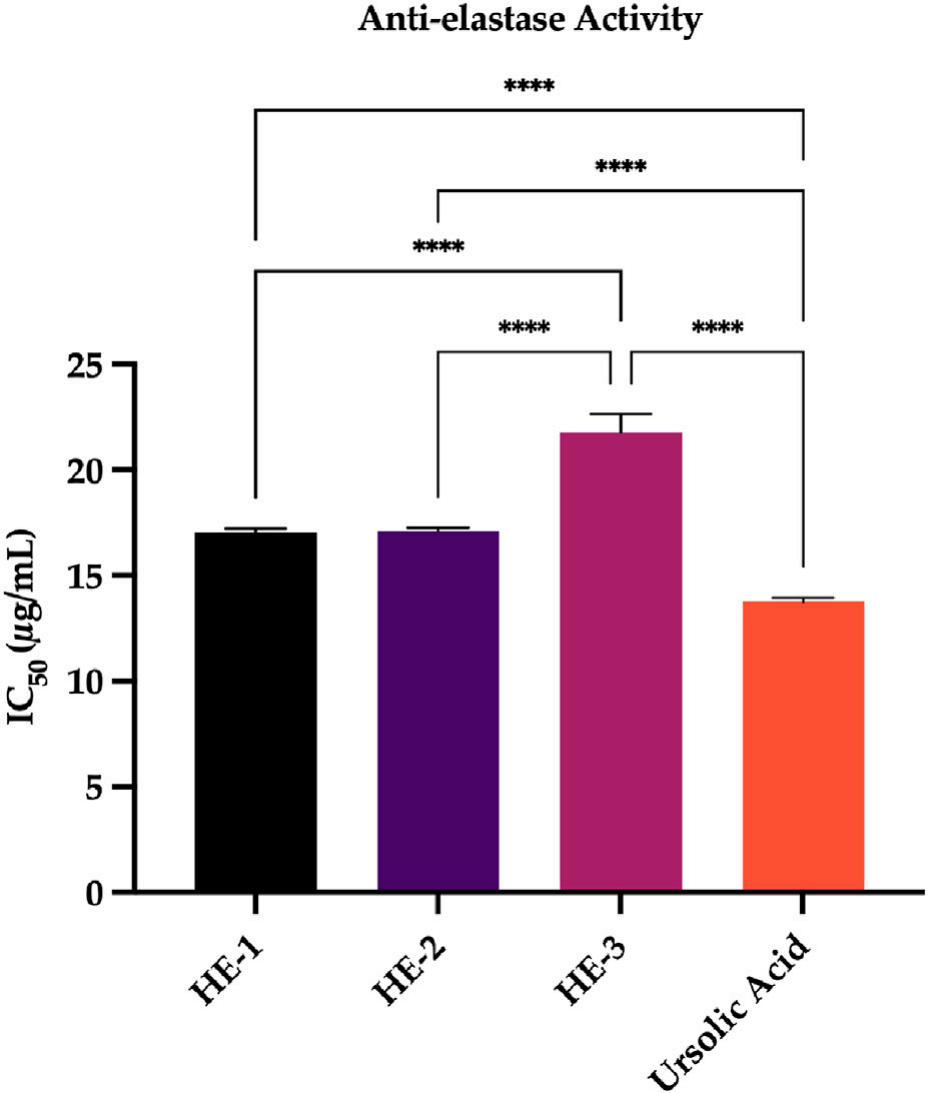
Anti-elastase Activity. Ursolic acid: standard compound. Statistical significance levels are indicated as follows: (^****^) P≤0.0001 .

In contrast, the HE extracts demonstrated moderate elastase inhibitory activity, with ${\text{IC}}_{50}$ values of 17.04 ± 0.18 μ g/mL, 17.12 ± 0.14 μ g/mL, and 21.79 ± 0.84 μ g/mL for HE-1, HE-2, and HE-3, respectively. However, these values were slightly higher than that of the standard inhibitor ursolic acid, which exhibited an ${\text{IC}}_{50}$ of 13.77 ± 0.17 μ g/mL. The moderate elastase inhibition observed suggests that while the extracts contain metabolites capable of modulating elastase activity, they are less potent compared to ursolic acid. This differential enzyme inhibitory profile may be attributed to the specific chemical composition of the extracts and their selective interactions with distinct enzyme active sites (Figure 6).

Although this study does not include primary ethnobotanical fieldwork, the selection of *H. empetrifolium* subsp. *empetrifolium* was based on its reported traditional use in the literature. The work aligns with the core principles of the Four Pillars of Best Practice in Ethnopharmacology (Heinrich et al., 2020), including proper taxonomic identification (with voucher specimen), use of pharmacologically relevant *in vitro* models, detailed phytochemical profiling, and transparent data reporting. The antifungal and enzyme inhibition assays were selected to reflect plausible therapeutic mechanisms relevant to the species’ ethnomedical context.

## 5 Conclusion

In conclusion, our study demonstrates that *H. empetrifolium* subsp. *empetrifolium* extracts exhibit significant antifungal activity against clinically relevant *Candida* strains, underscoring their potential as alternative therapeutic agents for fungal infections, their robust antifungal efficacy aligns with the pressing need for novel, effective antifungal agents.

Moreover, the extracts displayed potent α -Glucosidase inhibitory activity indicating promising antidiabetic potential. Importantly, the anti-cholinesterase assays revealed that the extracts, particularly HE-1, possess strong inhibitory effects against both acetylcholinesterase and butyrylcholinesterase, comparable to or better than standard inhibitors, thereby highlighting their potential in addressing neurodegenerative and mood-related disorders.

Phytochemical analyses via HPLC-DAD and LC-HR/MS further revealed notable variations in the profiles of key antioxidants, flavonoids, and phenolic metabolites among the samples. These differences, likely resulting from variations in collection locations and environmental conditions, emphasize the impact of growth conditions on the chemical composition and bioactivity of *H. empetrifolium* subsp. *empetrifolium*. Although lipophilic metabolites such as hypericin, pseudohypericin, and hyperforin were not detected, the presence of other bioactive phenolic constituents appears to underpin the diverse therapeutic properties observed.

Building upon these promising findings, future research should focus on the targeted isolation and structural elucidation of the most active constituents within *H. empetrifolium* subsp. *empetrifolium* extracts, employing advanced spectroscopic and chromatographic techniques. Moreover, standardized extraction protocols must be developed to ensure reproducibility and consistency in bioactivity, which are essential for translational applications. Given the demonstrated *in vitro* efficacy, comprehensive *in vivo* studies are crucial to assess pharmacokinetics, bioavailability, and potential toxicity profiles. In parallel, mechanistic investigations at the molecular level—particularly concerning enzyme inhibition and antifungal pathways—would provide deeper insights into the modes of action.

On the other hands, the notable chemical variations observed among samples collected from different geographical locations strongly suggest that environmental conditions significantly influence the phytochemical composition and, consequently, the bioactivity of *H. empetrifolium* subsp. *empetrifolium*. Therefore, future studies should include broader sampling from diverse habitats to identify the most favorable ecological conditions or specific locations associated with the highest concentration of bioactive metabolites. This approach would not only optimize raw material sourcing but also support conservation strategies and sustainable use of medicinal plant resources. Finally, the formulation of these extracts into biocompatible delivery systems could pave the way for novel phytopharmaceuticals and cosmeceuticals targeting fungal infections, metabolic disorders, and neurodegenerative diseases.

## Data Availability

The original contributions presented in the study are included in the article/Supplementary Material, further inquiries can be directed to the corresponding author.
