# Supplementary material for: Hypericum empetrifolium subsp. empetrifolium: an assessment of its antifungal, antidiabetic, anti-aging, and neuroprotective potential
Source: Front Pharmacol. 2025 Jul 7;16:1618761. doi: 10.3389/fphar.2025.1618761 (PMC12277336; doi:10.3389/fphar.2025.1618761)
Supplement: Supplementary file 1 [file DataSheet1.pdf]

frontiers

Supplementary Material

1 SUPPLEMENTARY TABLES AND FIGURES

1.1 Tables

Table S1. Calibration results and compound concentrations in samples

Compound	Retention Time (min)	Calibration Equation	r^2	Samples*		
				HE-1** (μg/mL)	HE-2** (μg/mL)	HE-3** (μg/mL)
Pseudohypericin	3.06	$Y = 1.689016 \times 10^{-8}X + 5.969209 \times 10^{-5}$	0.999957	Nd	Nd	Nd
Hypericin	6.58	$Y = 2.067168 \times 10^{-8}X + 2.490926 \times 10^{-5}$	0.999947	Nd	Nd	Nd
Hyperforin	24.62	$Y = 1.298948 \times 10^{-7}X - 8.150133 \times 10^{-4}$	0.999192	Nd	Nd	Nd

*: Values are means of three parallel measurment ± Standard deviation, $n = 3$ ($p < 0.05$).
**: *H. empetrifolium* subsp. *empetrifolium* extracts.

Table S2. LC-HR/MS analysis results of extracts

Compound	Samples*		
	HE-1** (μg/mL)	HE-2** (μg/mL)	HE-3** (μg/mL)
(-)-Epicatechin	Nd	75.881 ± 2.405	46.708 ± 1.481
(-)-Epigallocatechin	161.370 ± 4.986	74.253 ± 2.294	285.744 ± 8.830
(-)-Epigallocatechin gallate	86.167 ± 3.240	96.588 ± 3.632	89.018 ± 3.347
3'-O-Methyl quercetin	Nd	0.153 ± 0.006	Nd
Apigenin	0.089 ± 0.003	0.065 ± 0.002	0.106 ± 0.003
Ascorbic acid	52.503 ± 2.069	35.303 ± 1.391	49.879 ± 1.965
Chlorogenic acid	10.963 ± 0.393	2.184 ± 0.078	10.950 ± 0.392
Chrysin	0.012 ± 0.001	0.066 ± 0.002	Nd
Chrysoeriol	0.152 ± 0.003	0.129 ± 0.003	Nd
Dihydrokaempferol	0.725 ± 0.021	Nd	Nd
Ellagic acid	37.413 ± 1.571	64.848 ± 2.724	21.655 ± 0.910
Emodin	0.034 ± 0.002	0.008 ± 0.0003	0.019 ± 0.001
Fumaric acid	1021.486 ± 29.419	918.493 ± 26.453	1450.265 ± 41.768
Hispidulin 7-glucoside	75.892 ± 2.588	148.829 ± 5.075	Nd
Hyperoside	150.715 ± 5.215	223.164 ± 7.722	1624.240 ± 56.199
Kaempferol	Nd	0.081 ± 0.003	0.643 ± 0.023
Myricetin	4.262 ± 0.178	6.153 ± 0.257	1.816 ± 0.076
Naringenin	2.067 ± 0.087	1.295 ± 0.054	0.753 ± 0.032
p-Coumaric acid	47.567 ± 1.575	34.578 ± 1.145	Nd
Pyrogallol	1.228 ± 0.055	1.032 ± 0.046	1.009 ± 0.045
Quercetin	6.444 ± 0.190	6.376 ± 0.188	6.638 ± 0.196
Quercitrin	5.840 ± 0.221	6.236 ± 0.236	2997.251 ± 113.296
Rutin	10009.532 ± 307.293	11444.962 ± 351.360	648.337 ± 19.904
Salicylic acid	3.662 ± 0.069	5.658 ± 0.107	2.780 ± 0.052
Senecionine N-oxide	0.128 ± 0.005	Nd	Nd
Vanillic acid	264.615 ± 9.235	119.417 ± 4.168	162.998 ± 5.689

* Values are means of three parallel measurements ± standard deviation, $n = 3$ ($p < 0.05$).
** *H. empetrifolium* subsp. *empetrifolium* extracts.

Table S3. Determination of total phenolic and flavonoid content

**Samples**	**Total Phenolic Content** (μg PEs/mg extract)**	**Total Flavonoid Content** (μg QEs/mg extract
HE-1****	86.32 ± 1.91	36.81 ± 0.30
HE-2****	49.67 ± 1.15	43.97 ± 0.38
HE-3****	79.80 ± 1.61	54.07 ± 0.92

* Values expressed are means ± standard deviation of three parallel measurements ($p < 0.05$).
** PEs: pyrocatechol equivalents (y = 0.0307x + 0.048, R^2 = 0.9940).
*** QEs: quercetin equivalents (y = 0.0331x + 0.0855, R^2 = 0.9955).
**** *H. empetrifolium* subsp. *empetrifolium* extracts.

Table S4. Chemical tests assessing radical scavenging capacity results

**Plant Sample**	**Concentration (μg/mL)**	**DPPH (% Inhibition)**	**ABTS (% Inhibition)**	**CUPRAC (Absorbance)**
HE-1**	10	37.84 ± 0.76	78.01 ± 2.50	0.364 ± 0.11
	25	74.40 ± 2.23	89.05 ± 0.39	0.709 ± 0.02
	50	80.44 ± 0.10	89.22 ± 0.30	1.276 ± 0.04
	100	82.60 ± 0.57	88.53 ± 0.25	2.103 ± 0.11
HE-2**	10	45.84 ± 0.51	75.05 ± 2.13	0.356 ± 0.02
	25	76.24 ± 0.75	88.87 ± 0.54	0.606 ± 0.01
	50	77.87 ± 0.73	89.05 ± 0.25	0.969 ± 0.03
	100	79.13 ± 0.47	88.53 ± 0.50	1.877 ± 0.12
HE-3**	10	33.56 ± 0.28	52.68 ± 1.16	0.357 ± 0.05
	25	58.33 ± 1.39	88.43 ± 0.39	0.69 ± 0.08
	50	72.24 ± 1.82	87.13 ± 2.64	1.729 ± 0.36
	100	77.42 ± 1.76	88.08 ± 0.45	1.712 ± 0.12
BHA***	10	62.00 ± 0.90	63.94 ± 0.18	0.838 ± 0.03
	25	71.86 ± 0.76	78.23 ± 1.08	1.647 ± 0.18
	50	74.48 ± 0.35	87.41 ± 0.16	2.753 ± 0.09
	100	75.23 ± 0.23	87.62 ± 0.00	3.769 ± 0.05
α-TOC***	10	63.28 ± 3.28	65.39 ± 2.37	0.391 ± 0.01
	25	73.51 ± 4.14	83.47 ± 0.98	0.904 ± 0.00
	50	76.94 ± 0.71	85.89 ± 2.62	1.563 ± 0.09
	100	78.07 ± 0.50	88.89 ± 1.05	2.460 ± 0.09
BHT***	10	39.28 ± 1.03	76.35 ± 0.78	0.652 ± 0.01
	25	60.68 ± 4.18	88.00 ± 2.49	1.211 ± 0.18
	50	69.14 ± 1.84	88.96 ± 0.13	2.040 ± 0.31
	100	76.17 ± 0.32	89.13 ± 0.13	3.559 ± 0.27

* Values expressed are means ± standard deviation of three parallel measurements ($p < 0.05$).
** *H. empetrifolium* subsp. *empetrifolium* extracts.
*** Standard antioxidants.

Table S5. Antimicrobial activity results of extracts

Strains	Samples			Positive Control (μg/mL)
	HE-1* (μg/mL)	HE-2* (μg/mL)	HE-3* (μg/mL)	
**Gram (-) bacterial strains**				
P. aeruginosa ATCC 27853	NA	NA	625	Ceftazidime 2.4
E. coli ATCC 25922	NA	NA	NA	Cefuroxime-Na 4.9
K. pneumoniae ATCC 4352	NA	NA	NA	Cefuroxime-Na 4.9
P. mirabilis ATCC 14153	NA	NA	NA	Cefuroxime-Na 2.4
**Gram (+) bacterial strains**				
S. aureus ATCC 29213	625	1250	1250	Cefuroxime-Na 1.2
S. epidermidis ATCC 12228	NA	NA	NA	Cefuroxime-Na 9.8
E. faecalis ATCC 29212	1250	1250	1250	Amikacin 128
**Yeast strains**				
C. albicans ATCC 10231	156.2	156.2	156.2	Clotrimazole 4.9
C. parapsilosis ATCC 22019	78.12	156.2	78.12	Amphotericin B 1
C. tropicalis ATCC 750	78.12	78.12	78.12	Amphotericin B 0.5

* *H. empetrifolium* subsp. *empetrifolium* extracts.

Table S6. Enzyme inhibition activities results

Samples	IC_{50} Values μg/mL					
	AChE	BChE	Tyrosinase	α-Glucosidase	Elastase	Hyaluronidase
HE-1	8.16 ± 0.39[a]	2.46 ± 0.02[a]	110.02 ± 0.91[a]	26.2 ± 1.52[a]	17.04 ± 0.18[a]	NA
HE-2	17.55 ± 0.82[b]	13.46 ± 0.42[b]	101.76 ± 0.35[b]	30.27 ± 1.04[a]	17.12 ± 0.14[a]	NA
HE-3	42.09 ± 1.48[c]	26.42 ± 0.86[c]	98.56 ± 1.31[c]	30.91 ± 0.51[a]	21.79 ± 0.84[b]	NA
Galantamine**	8.53 ± 0.20[a]	38.66 ± 0.49[d]	–	–	–	–
Kojic acid***	–	–	21.70 ± 0.97[d]	–	–	–
Acarbose****	–	–	–	676.5 ± 10.5[b]	–	–
Ursolic acid*****	–	–	–	–	13.77 ± 0.17[c]	78.62 ± 1.46

** Standard inhibitor for AChE and BChE.
*** Standard inhibitor for Tyrosinase.
**** Standard inhibitor for α-Glucosidase.
***** Standard inhibitor for Elastase and Hyaluronidase.
Superscript letters indicate significant differences among samples for each assay independently ($p < 0.05$, one-way ANOVA with Tukey's post hoc test).

1.2 Figures

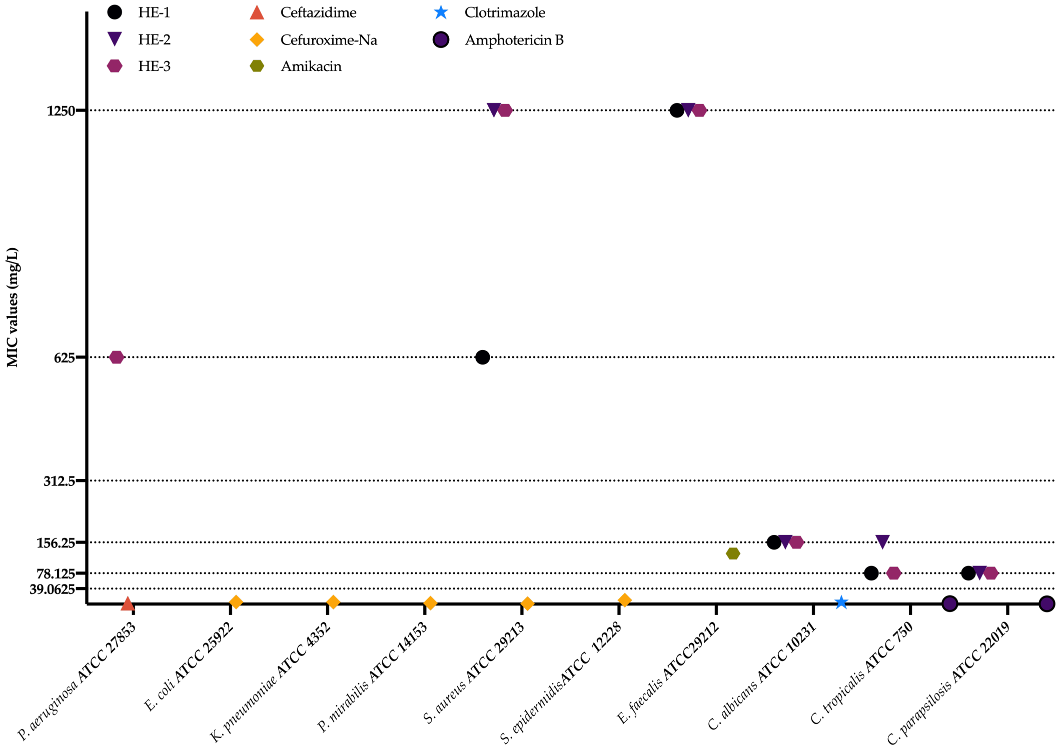

Figure S1. Antimicrobial activity assays

HE-1,2,3: extract codes.

Ceftazidime, Cefuroxime-Na, Amikacin, Clotrimazole, and Amphotericin B: standard antimicrobials.

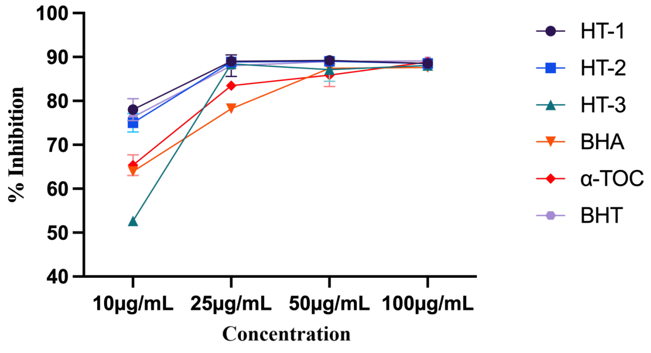

Figure 2a. ABTS Cation Scavenging Capacity

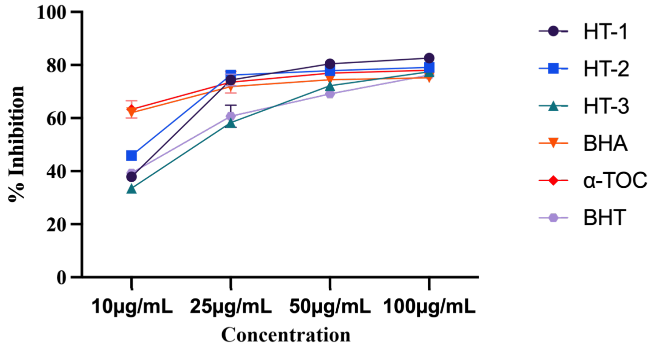

Figure 2b. DPPH Free Radical Scavenging Capacity

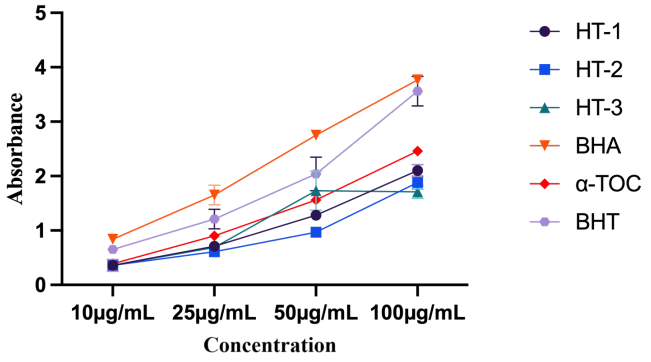

Figure 2c. CUPRAC

Figure 2. Chemical tests assessing radical scavenging capacity results

HE-1,2,3: extract codes.
BHA, α-TOC, and BHT: standard antioxidants
Statistical significance levels are indicated as follows: (**) $P \leq 0.01$; (***) $P \leq 0.001$; (****) $P \leq 0.0001$.

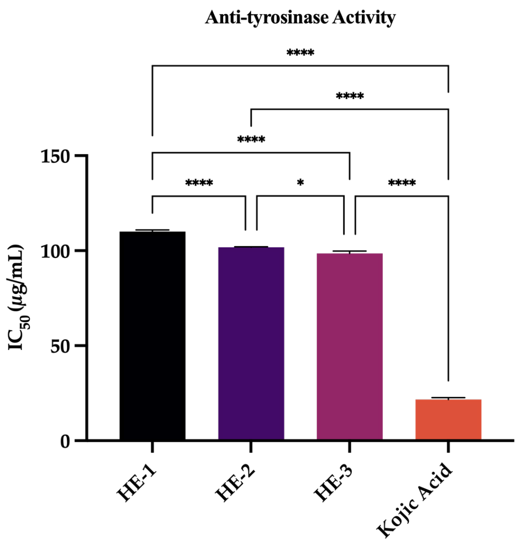

Figure S3. Anti-tyrosinase activity

Kojic acid: standard inhibitor.
Statistical significance levels are indicated as follows: (*) $P \leq 0.05$; (***) $P \leq 0.001$; (****) $P \leq 0.0001$.

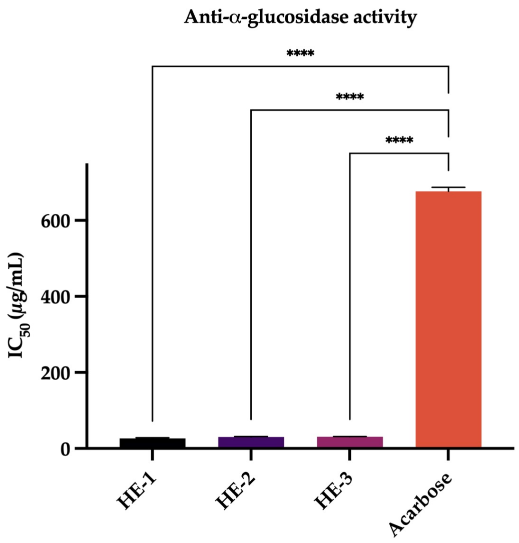

Figure S4. anti-α-Glucosidase activity

Galantamine: standard inhibitor.
Statistical significance levels are indicated as follows: (****) $P \leq 0.0001$.

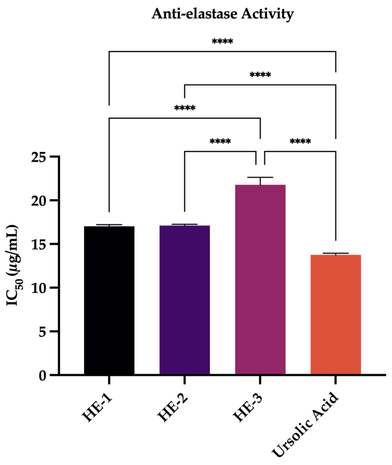

Figure S5. Anti-elastase Activity

Ursolic acid: standard inhibitor.
Statistical significance levels are indicated as follows: (****) $P \leq 0.0001$.

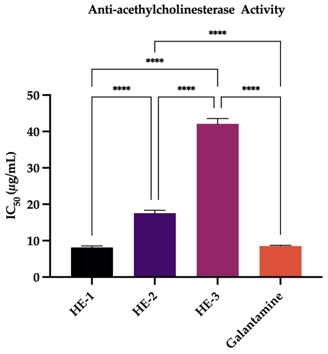

Figure 6a. Anti-acetylcholinesterase activity

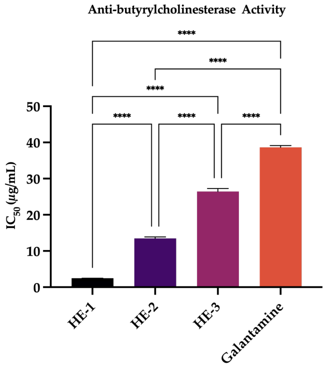

Figure 6b. Anti-butyrylcholinesterase activity

Figure 6. Anti-cholinesterase activity results

HE-1,2,3: extract codes.
Galantamine, kojic acid, acarbose, and ursolic acid: standard inhibitors.
Statistical significance levels are indicated as follows: (*) $P \leq 0.05$;(**) $P \leq 0.01$; (***) $P \leq 0.001$; (****) $P \leq 0.0001$.

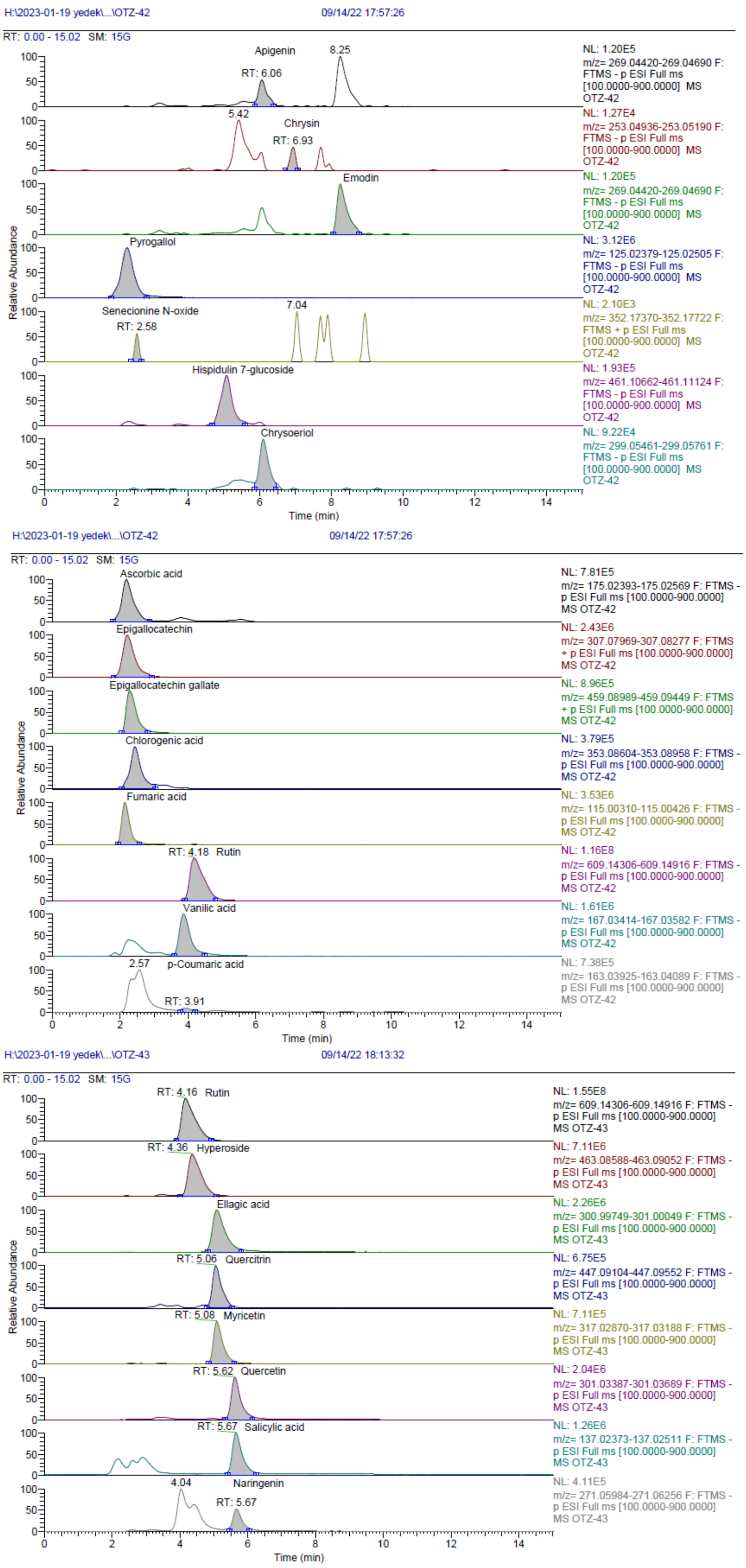

Figure S7. LC-HR/MS chromatograms of HE-1

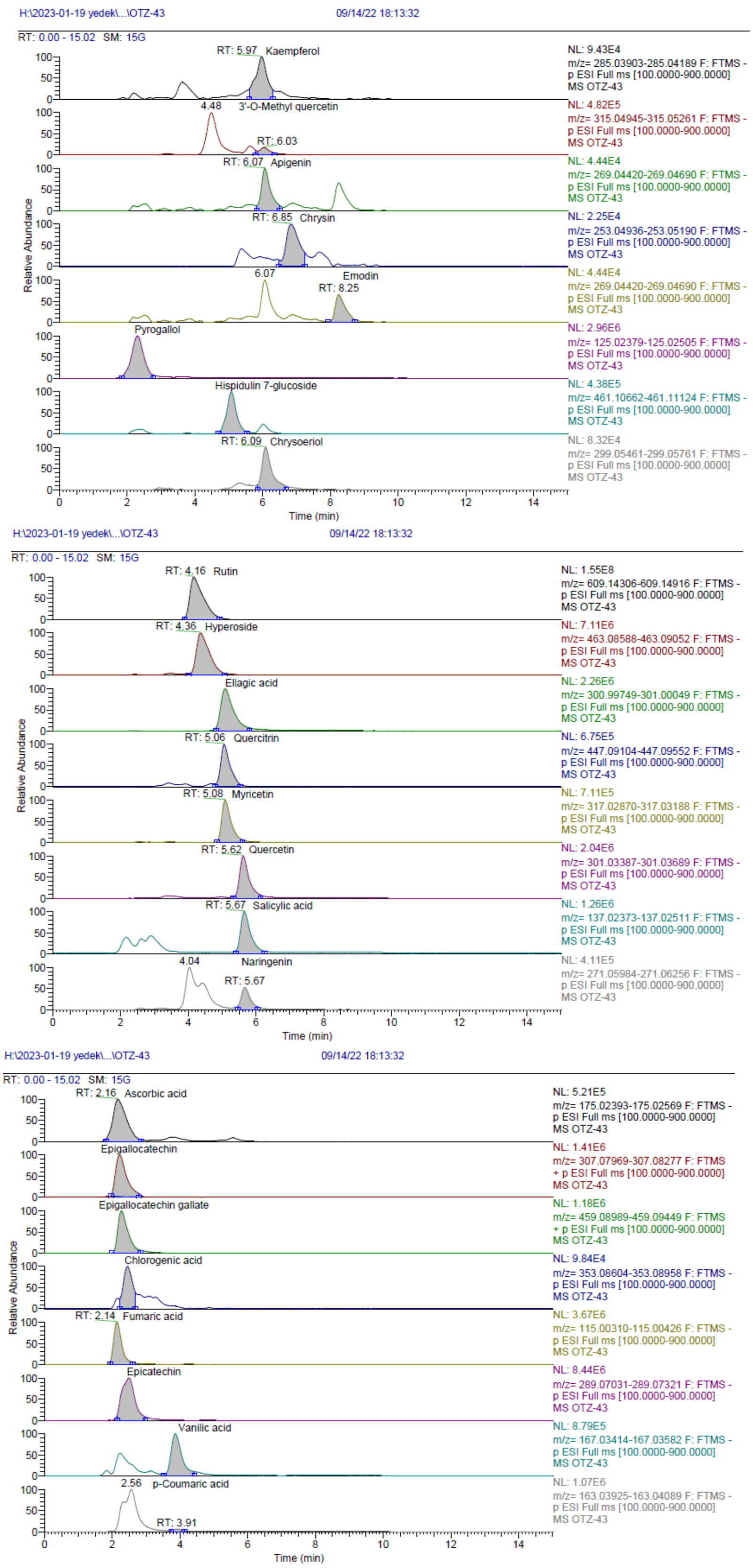

Figure S8. LC-HR/MS chromatograms of HE-2

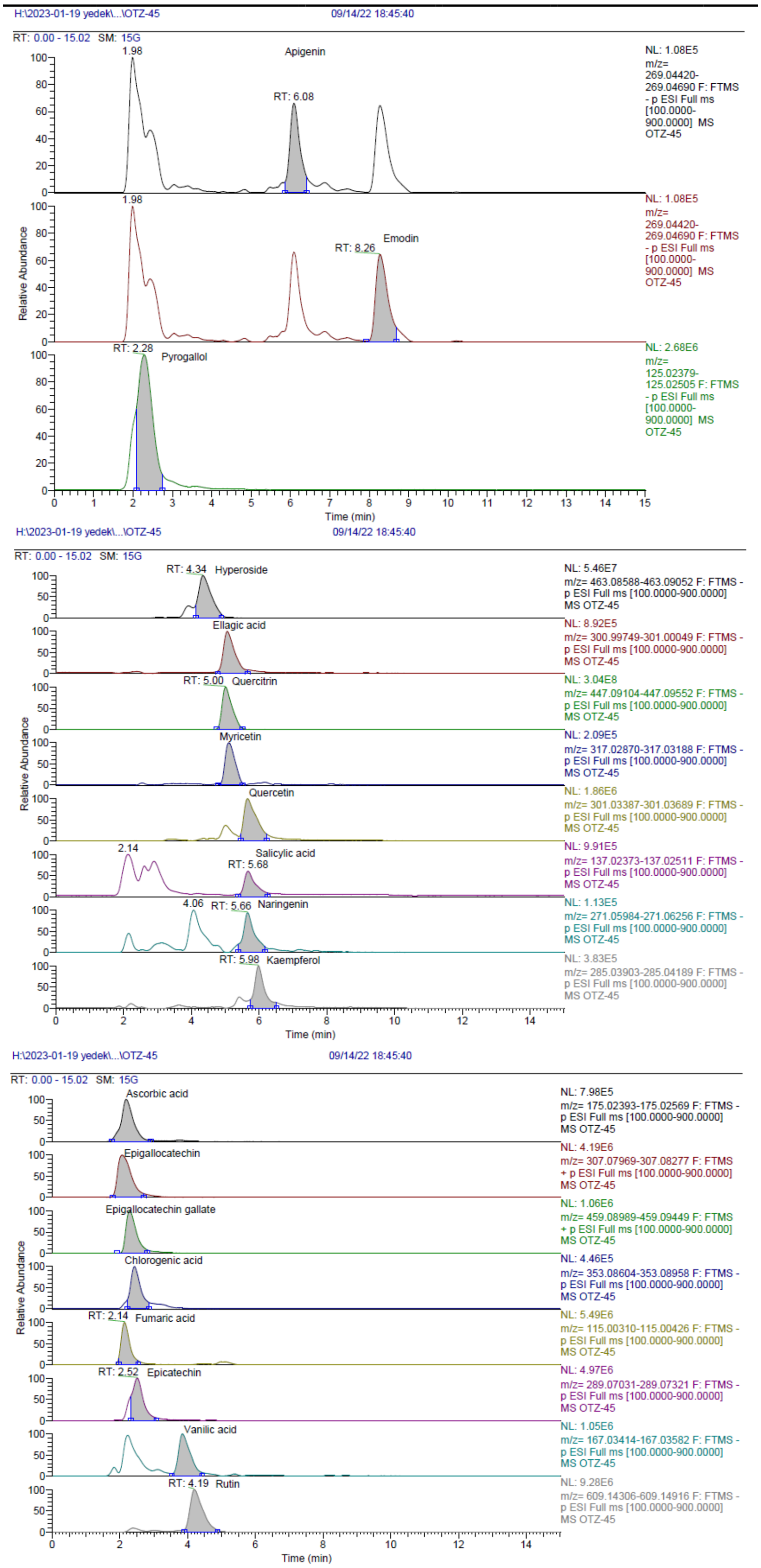

Figure S9. LC-HR/MS chromatograms of HE-3
